# Interfacial Engineering Strategies of Self-Assembled Monolayers for Inverted Perovskite Solar Cells

**DOI:** 10.3390/nano16160989

**Published:** 2026-08-11

**Authors:** Yong Ge, Kelei Wang, Runnan Yu, Zhan’ao Tan

**Affiliations:** Beijing Advanced Innovation Center for Soft Matter Science and Engineering, Beijing University of Chemical Technology, Beijing 100029, China; 15099155115@163.com (Y.G.); w1370348740@163.com (K.W.)

**Keywords:** inverted perovskite solar cells, self-assembled monolayers, buried interfaces, hole-selective contacts, interfacial engineering

## Abstract

Inverted perovskite solar cells (PSCs), or p-i-n PSCs, have become increasingly attractive for high-performance perovskite photovoltaics owing to their low-temperature processability, reduced hysteresis, flexible-substrate compatibility and suitability for perovskite/silicon tandem architectures. The buried interface is central to charge extraction, energy-level alignment, perovskite crystallization and operational stability, and is therefore a key determinant of device performance. Self-assembled monolayers (SAMs) are molecularly thin and offer negligible parasitic absorption, tunable interfacial energetics, low material loading and high structural designability, making them attractive alternatives to conventional organic Hole Transport Layers and effective hole-selective contacts in inverted PSCs. This review examines molecular design principles and interfacial engineering strategies for SAMs in inverted PSCs, focusing on the phosphonic acid carbazole (PACz) family, substituent and terminal-group engineering, and emerging conjugated backbones. We then summarize how SAMs regulate buried interfaces through energy-level alignment, defect passivation, crystallization control and stability enhancement. We further highlight emerging interface strategies, including co-assembled SAMs, amorphous SAMs, polymerized or crosslinked SAMs and molecular hybrid interfaces, and discuss how data-driven molecular screening may accelerate future SAM discovery. Finally, we discuss outstanding challenges in SAM formation, large-area uniformity, in situ and operando characterization, and data-driven molecular design, and provide perspectives on the use of SAMs in efficient, durable and scalable inverted PSCs.

## 1. Introduction

Driven by climate change and the growing demand for low-carbon energy, photovoltaics are expected to play a central role in future renewable-energy systems [1]. Besides crystalline silicon, emerging photovoltaic systems-including solution-processable dye-sensitized, organic and colloidal quantum-dot solar cells, as well as III-V nanowire solar cells with nanoscale light-management and band-engineering capabilities-have been widely investigated [2,3,4,5,6,7,8]. Nevertheless, metal halide perovskites remain particularly competitive because they combine strong light absorption, long carrier diffusion lengths, low-temperature processability and rapidly increasing device efficiencies, with inverted PSCs and perovskite/silicon tandem devices showing particular promise for next-generation photovoltaics [6]. Organic-inorganic metal halide perovskites have emerged as a promising class of light-harvesting materials for solar cell applications owing to their strong light absorption, long carrier diffusion lengths and compatibility with low-cost solution processing. Since their first photovoltaic application in 2009, the PCE of single-junction PSCs has increased from an initial 3.8% to a certified 27.31% [9,10]. As device efficiencies continue to increase, interfacial non-radiative recombination has become an increasingly important loss pathway, particularly as bulk defect management improves [11]. High-performance PSCs are mainly based on two device architectures: the regular n-i-p structure and the inverted p-i-n structure. Early high-efficiency devices were predominantly based on the regular n-i-p architecture, but this configuration is often associated with pronounced hysteresis, interfacial ion accumulation and processing complexity related to high-temperature treatments. By contrast, the inverted p-i-n architecture offers low-temperature processability, reduced hysteresis, excellent compatibility with flexible substrates and greater suitability for large-area fabrication, and has therefore attracted increasing attention in recent years [12]. The rise in perovskite/silicon tandem solar cells has further strengthened the appeal of the inverted architecture, in which low-temperature processability and tunable interfaces make p-i-n structures particularly suitable for high-efficiency tandem devices [13]. As a result, p-i-n PSCs are becoming a key platform for next-generation single-junction and tandem perovskite photovoltaics.

**Figure 1:**
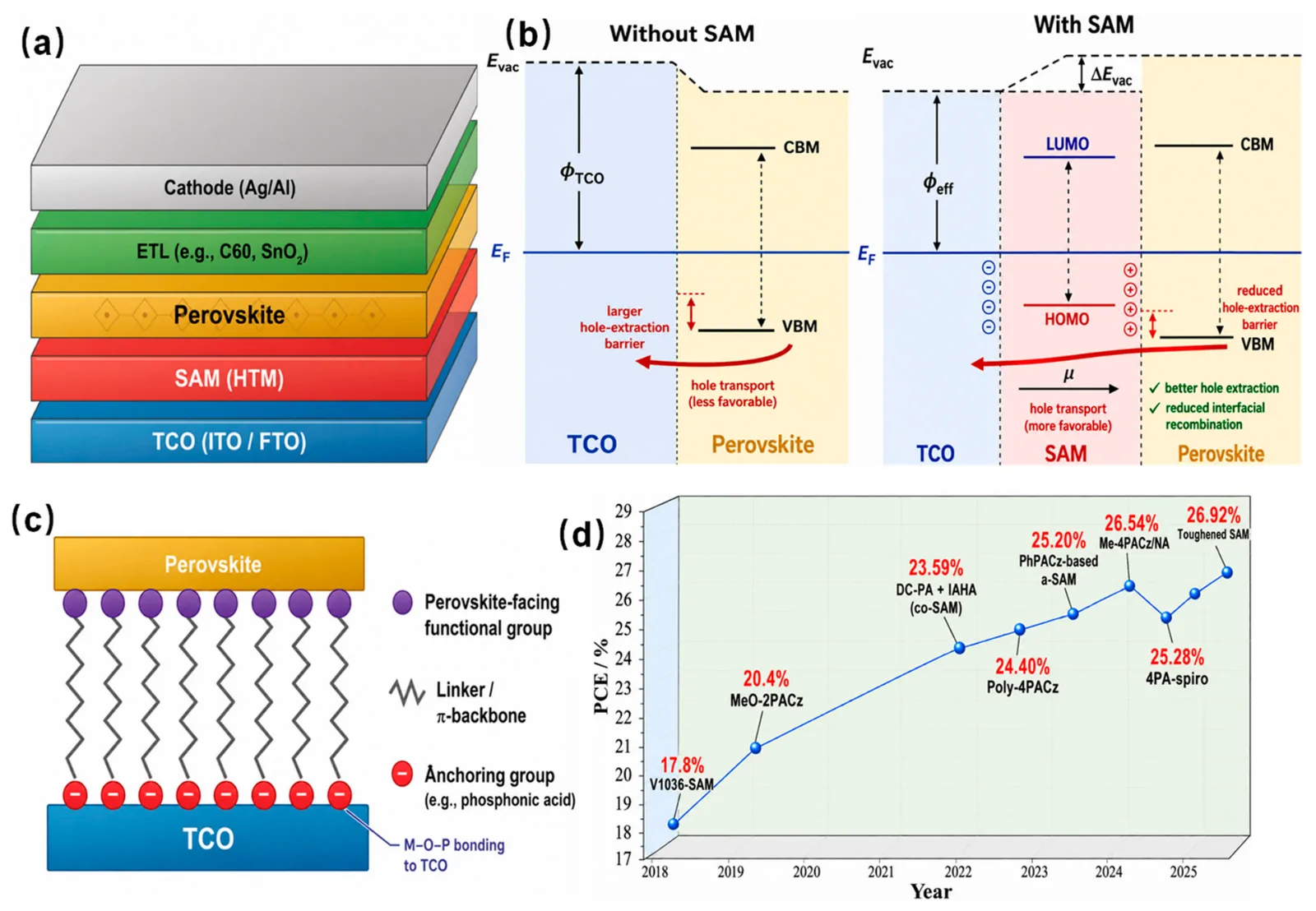
Overview of SAM-based buried-interface engineering in inverted perovskite solar cells. (**a**) Typical inverted p-i-n device structure using SAM as the hole-selective contact between TCO and perovskite. (**b**) Energy-level alignment without and with SAM modification. SAM-induced interfacial dipoles tune the TCO work function, reduce the hole-extraction barrier and facilitate hole transport. (**c**) Schematic molecular configuration of SAMs at the TCO/perovskite interface, showing the anchoring group, linker/π-backbone and perovskite-facing functional group. (**d**) Representative development timeline of SAM-based inverted PSCs with selected reported PCE values [16,18,23,29,30,31,32,33,34]. These efficiencies are shown as literature reference points rather than directly comparable records.

In p-i-n PSCs, the bottom Hole Transport Layer (HTL) is deposited on the Transparent Conductive Oxide (TCO) substrate and serves as the interfacial layer between the electrode and the perovskite absorber (Figure 1a–c). Its properties are critical not only for hole extraction and energy-level alignment but also for perovskite film formation. Conventional inverted devices have commonly relied on poly(3,4-ethylenedioxythiophene):poly(styrenesulfonate) (PEDOT:PSS) and poly[bis(4-phenyl)(2,4,6-trimethylphenyl)amine] (PTAA) as HTL; however, the acidity and hygroscopic nature of PEDOT:PSS can corrode the Indium Tin Oxide (ITO) electrode and accelerate interfacial degradation [14]. Although PTAA has enabled high device efficiencies, its high cost, batch-to-batch variability and sensitivity to processing conditions remain important limitations [15]. Therefore, self-assembled monolayers (SAMs) have emerged as key hole-selective contacts for inverted PSCs [16]. Compared with conventional HTLs such as PEDOT:PSS and PTAA, SAMs can function as efficient charge-selective contacts in p-i-n PSCs [17]. Their molecularly thin configuration minimizes parasitic absorption and avoids the need for thick organic transport layers [18]. Their low material loading is advantageous for scalable and cost-effective processing [19], while their modular molecular structures provide substantial tunability over interfacial energetics and chemistry [20]. Representative PACz molecules, including [2-(9H-carbazol-9-yl)ethyl]phosphonic acid (2PACz), [2-(3,6-dimethoxy-9H-carbazol-9-yl)ethyl]phosphonic acid(MeO-2PACz) and [4-(3,6-dimethyl-9H-carbazol-9-yl)butyl]phosphonic acid(Me-4PACz), use phosphonic acid anchoring groups to bind to oxide electrodes such as ITO or NiO_x_, and their conjugated carbazole backbones enable efficient low-loss hole-selective contacts [18,21,22]. In recent years, inverted PSCs with SAM-engineered buried interfaces have achieved certified efficiencies exceeding 26%, demonstrating that SAMs have become a key interfacial engineering strategy for advancing high-performance inverted PSCs [23]. More importantly, SAMs are no longer viewed merely as substitutes for conventional HTLs, but rather as molecular-scale platforms for engineering buried interfaces [24]. By tailoring molecular dipoles, interfacial chemical interactions and surface energy, SAMs can regulate electrode work function, charge extraction, interfacial recombination, perovskite nucleation and growth, and interfacial stability [20,25,26,27]. Therefore, elucidating the molecular design principles of SAMs and their roles in buried-interface regulation is essential for developing inverted PSCs with higher efficiency, improved operational stability and greater compatibility with scalable fabrication [28].

Although SAMs have substantially advanced the performance of inverted PSCs, most studies have focused on molecular-structure optimization and efficiency enhancement. By contrast, the mechanisms governing SAM formation, buried-interface regulation and long-term interfacial stability remain insufficiently understood, limiting the rational design of efficient, stable and scalable inverted PSCs [35,36]. However, the adsorption, molecular orientation, surface coverage and temporal evolution of SAMs on practical oxide substrates remain insufficiently understood [37,38]. For example, certain PACz-based SAMs can aggregate or form spatially non-uniform coverage under practical processing conditions, which compromises buried-interface uniformity and device reproducibility [39]. Therefore, understanding how SAMs form on oxide substrates and how their formation characteristics correlate with interfacial properties has become an important research focus.

In addition, the long-term stability of SAM-based interfaces remains insufficiently understood. Although interfacial-layer stability is critical for the long-term operation of inverted PSCs, how SAMs maintain their structural integrity under thermal, light and damp-heat stress, and how their degradation contributes to buried-interface failure and device performance loss, remain to be clarified [40]. From a practical perspective, large-area fabrication and process reproducibility remain major barriers to the practical deployment of SAM-based interfaces. Because SAM deposition is highly sensitive to spin-coating parameters, solvent environment and substrate conditions, batch-to-batch variation can readily arise during device fabrication [41,42]. Although scalable processes such as blade coating, slot-die coating and thermal evaporation are being explored, their applicability to uniform, reproducible and high-throughput SAM deposition has yet to be fully established [43]. Recent reviews have provided valuable summaries of SAM-based hole-selective contacts, molecular structures, chemical synthesis and their applications in perovskite photovoltaics. However, molecular design, self-assembly behaviour on practical oxide substrates, coupled buried-interface functions, interfacial failure and scalable processing have often been discussed as relatively separate topics. Consequently, a unified understanding of how molecular modifications influence assembly behaviour and interfacial properties, and ultimately determine device efficiency, stability and reproducibility, remains limited. Against this background, this review integrates molecular design, self-assembly behaviour, buried-interface regulation and scalable processing within a structure–assembly–interface–device framework. We correlate anchoring groups, conjugated backbones and interfacial functional groups with molecular adsorption, orientation, surface coverage and packing on practical substrates. Energy-level alignment, selective carrier extraction, defect passivation, crystallization regulation and interfacial stabilization are further examined as mutually coupled rather than independent processes. In addition, co-assembled, amorphous and polymerized or crosslinked SAM strategies are compared in terms of functional complementarity, coverage integrity, processing tolerance, large-area uniformity and long-term reliability. Through this integrated analysis, this review aims to derive transferable design principles for efficient, stable and scalable SAM-based buried interfaces.

The remainder of this review is organized as follows. Section 2 discusses the molecular design principles of SAMs, including benchmark PACz systems, substituent and functional-group engineering, and emerging conjugated backbones. Section 3 examines the coupled mechanisms through which SAMs regulate buried interfaces, including energy-level alignment, carrier extraction, defect passivation, crystallization control and interfacial stabilization. Section 4 compares emerging interface-engineering strategies, including co-assembled, amorphous, and polymerized or crosslinked SAMs. Section 5 discusses the remaining challenges and future perspectives related to SAM formation, scalable processing, dynamic characterization and predictive molecular design. Finally, Section 6 summarizes the principal conclusions and future implications of this Review.

## 2. Molecular Design Principles of Sams

**Figure 2:**
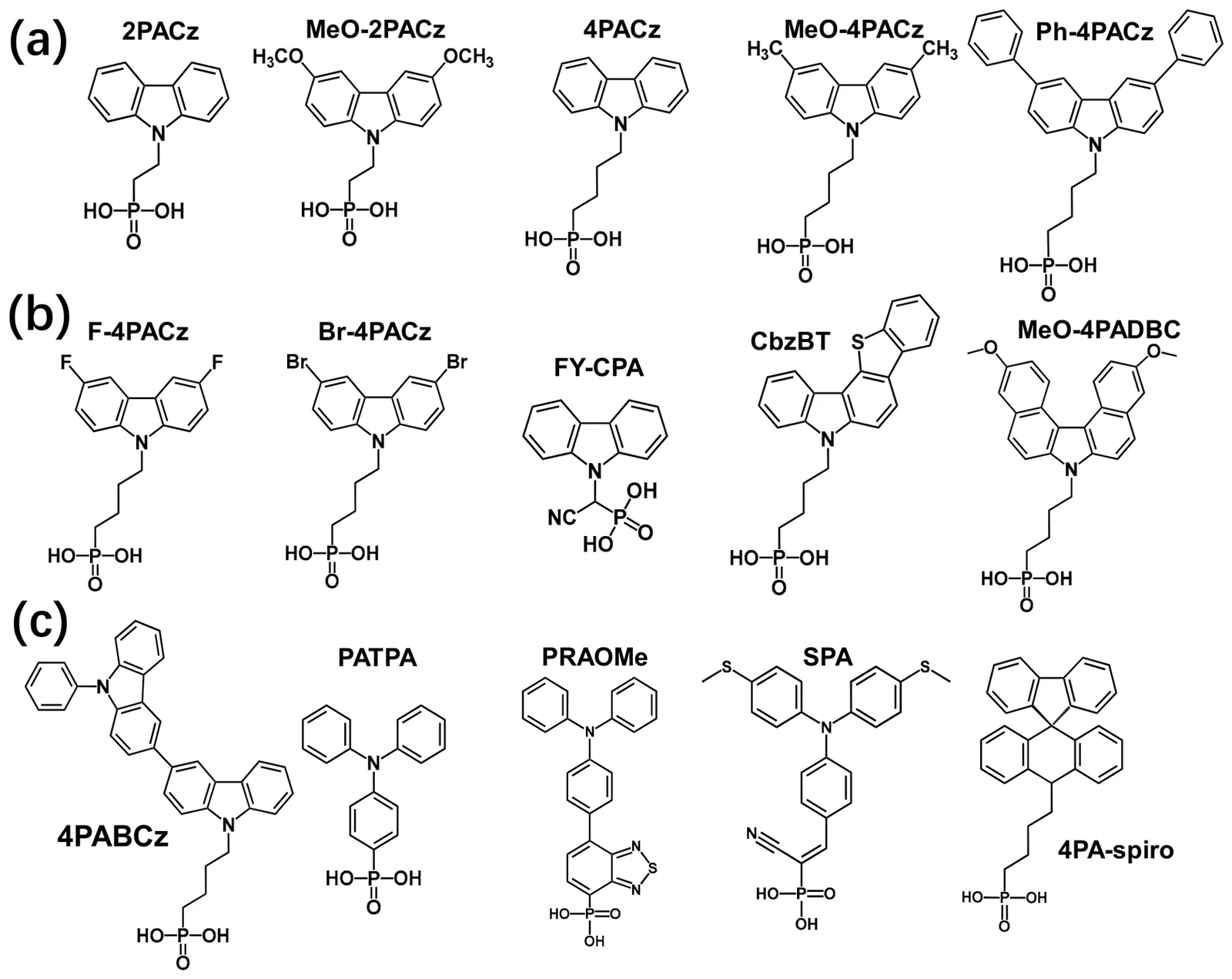
Representative molecular structures of SAM-forming molecules for inverted perovskite solar cells. (**a**) PACz-based benchmark SAMs. (**b**) Substituent- and interfacial functional-group-engineered SAMs for tuning dipoles, surface energy and interfacial interactions. (**c**) Emerging conjugated-backbone SAMs that expand the structural design space beyond conventional PACz molecules.

The distinctive interfacial functions of SAMs originate from their modular molecular architecture. In general, SAM-forming hole-selective molecules can be described using three key structural modules: an anchoring group, a π-conjugated backbone and interfacial functional groups or substituents [18]. In PACz-based systems, the phosphonic acid anchoring group binds to oxide electrodes; whereas, the carbazole backbone contributes to hole-selective transport, enabling the formation of conformal and low-loss molecular contacts [18,22]. Interfacial functional groups or substituents regulate molecular dipoles, surface polarity, wettability, molecular packing and interactions with the perovskite surface [36] (Figure 2). The resulting SAM coverage also depends on the oxide substrate; for example, ALD-grown NiO_x_ was shown to enhance SAM surface coverage [22]. Therefore, rational SAM design requires the coordinated optimization of anchoring chemistry, backbone structure and interfacial functional groups to construct ultrathin hole-selective contacts with stable adsorption, suitable energetics and good buried-interface compatibility.

### 2.1. The Pacz Family as Benchmark Sams

The PACz family, consisting of phosphonic-acid-functionalized carbazole molecules, is among the most extensively studied SAM systems in inverted PSCs [36]. PACz molecules typically adopt a modular design that integrates a phosphonic acid anchoring group, a carbazole-based π-conjugated backbone and functional substituents [20]. The phosphonic acid group binds to oxide surfaces such as ITO, FTO or NiO_x_, providing interfacial anchoring for the molecular layer, while the carbazole backbone contributes to efficient hole-selective transport [18]. Functional substituents can further tune the molecular electrostatic potential, energy levels and dipole characteristics of PACz derivatives [44]. In addition to molecular structure, the electronic properties of carbazole-based SAMs on TCO substrates are influenced by the microstructure of the underlying electrode [21].

The work by Al-Ashouri and co-workers established carbazole-based phosphonic acid molecules, including 2PACz, MeO-2PACz and Me-4PACz, as effective ultrathin hole-selective contacts for single-junction and perovskite/silicon tandem devices. By demonstrating that an approximately 1 nm-thick SAM could enable efficient hole selectivity, this study laid the groundwork for the subsequent development of PACz-based SAMs for buried-interface engineering in inverted PSCs [18]. Subsequent work has moved beyond using PACz molecules merely as ultrathin hole-selective contacts, instead emphasizing the coordinated tuning of molecular dipoles, backbone substitution, linker length and interfacial assembly behaviour to optimize buried-interface properties [36].

Within the PACz family, 2PACz represents a prototype carbazole–phosphonic acid SAM, illustrating the early design principle of combining an oxide-binding anchoring group with a hole-selective conjugated backbone [18]. MeO-2PACz further extends this framework by introducing methoxy substituents onto the carbazole backbone, which can tune the molecular electronic structure, surface polarity and interfacial wettability. This molecule therefore provides a representative example of substituent engineering within PACz-based SAMs [44]. Me-4PACz further modifies this molecular framework by extending the alkyl linker and introducing methyl substituents on the carbazole backbone, thereby altering molecular conformation, packing and surface coverage. These features have made Me-4PACz one of the most widely used PACz-based SAMs in high-efficiency inverted PSCs [23].

Overall, the evolution from 2PACz to MeO-2PACz and Me-4PACz reflects a broader shift in PACz-based SAM design: from forming ultrathin hole-selective contacts to coordinating molecular electronic structure, surface properties and interfacial assembly. This transition has laid the groundwork for subsequent substituent engineering and conjugated-backbone diversification [20,36,44].

### 2.2. Substituent and Interfacial Functional-Group Engineering

Following the establishment of the PACz family, substituent and interfacial functional-group engineering has emerged as an important strategy for tailoring the electronic structure, molecular dipole, surface energy, packing behaviour and interfacial compatibility of SAMs. By introducing electronic, steric and polarity effects, substituents and terminal functional groups can modulate molecular dipoles, HOMO levels, surface energy and molecular packing, which may further influence the compatibility of SAMs with oxide substrates and perovskite precursor inks [36,44,45].

**Figure 3:**
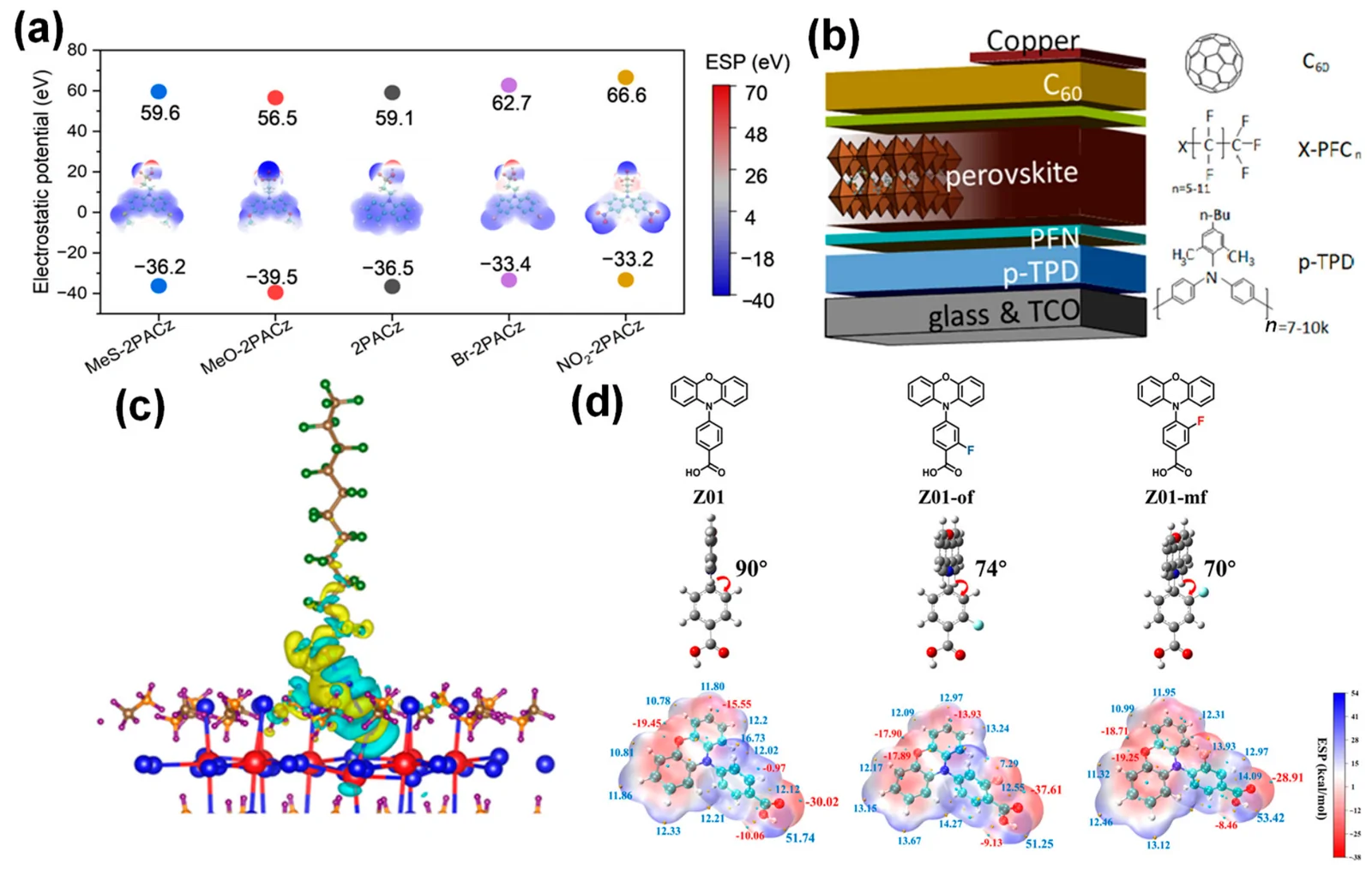
Substituent and fluorination engineering of SAMs for inverted perovskite solar cells. (**a**) Electrostatic potential distributions of MeS-2PACz, MeO-2PACz, 2PACz, Br-2PACz and NO_2_-2PACz, showing substituent-dependent modulation of molecular polarity and electrostatic potential. Reprinted with permission from Ref. [44]. Copyright 2025 Royal Society of Chemistry. (**b**,**c**) Device architecture and interfacial interaction of perfluorinated SAM-modified inverted PSCs. Reprinted with permission from Ref. [46]. Copyright 2020 American Chemical Society. (**d**) Molecular structures, optimized configurations and electrostatic potential distributions of Z01, Z01-of and Z01-mf, illustrating the effect of phenyl-linker fluorination on molecular conformation and surface electrostatic distribution. Reprinted with permission from Ref. [47]. Copyright 2026 American Chemical Society.

Early efforts to improve the performance of PACz-based SAMs frequently relied on substituent engineering. Huang et al. [44] systematically designed a series of 2PACz derivatives by introducing electron-donating and electron-withdrawing substituents, including MeS, MeO, Br and NO_2_ groups, onto the carbazole unit. These molecular modifications tune the electrostatic potential distribution and energy levels of the SAM molecules, thereby influencing the energy alignment between the NiO_x_ Hole Transport Layer and MAPbI_3_ perovskite as well as interfacial charge extraction. Among these derivatives, MeO-2PACz showed the most favourable energy-level alignment, enabling a maximum PCE of 21.67% and a T_80_ lifetime of 17 h under continuous UV illumination [44]. The electrostatic potential distributions shown in Figure 3a further illustrate that substituent engineering can regulate molecular polarity and interfacial dipole characteristics, providing a molecular basis for tuning SAM-mediated buried-interface energetics [44].

In addition to electron-donating substituents, electron-withdrawing substituent engineering has also been explored, with fluorination being a representative strategy. Representative fluorinated SAM strategies are summarized in Figure 3b–d, including perfluorinated SAM modification and phenyl-linker fluorination. Owing to the strong electron-withdrawing character and low surface energy associated with fluorinated groups, fluorination can tune molecular dipoles, surface energy and the interfacial chemical environment, thereby offering a useful route to modulate the interfacial properties of SAMs [36]. The perfluorinated SAMs reported by Wolff and co-workers were associated with improved efficiency and environmental stability in inverted PSCs, highlighting fluorinated terminal groups as an important molecular design strategy for tailoring SAM interfacial properties [46]. More recently, Liang et al. [47] designed phenoxazine-based SAMs with different phenyl linkers, namely Z01, Z01-of and Z01-mf, in which the carboxylic acid anchoring group is connected to the phenoxazine headgroup through non-fluorinated, ortho-fluorinated and meta-fluorinated phenyl spacers, respectively. Compared with Z01 and Z01-of, Z01-mf exhibited higher molecular planarity and a lower HOMO energy level; moreover, the meta-fluorine substituent weakened intermolecular hydrogen bonding involving the carboxyl anchoring group, which facilitated more compact molecular packing on ITO substrates. As a result, Z01-mf-based inverted PSCs achieved a fill factor of 84.43%, higher than those of Z01-of and Z01 devices. Taken together, these studies suggest that fluorination can tune not only molecular energetics and surface hydrophobicity but also molecular conformation, intermolecular interactions and packing density, thereby providing design opportunities for constructing more stable and efficient buried interfaces.

Beyond fluorinated substituents, cyano, carbonyl and heteroatom-containing functional groups, including sulfur- and selenium-containing motifs, have also been incorporated into SAM molecular design. These groups generally possess pronounced polarity or Lewis-basic character, which may help tune molecular dipoles, surface chemistry and interactions with the perovskite surface [36,48]. Such functional groups can interact with under-coordinated Pb^2+^ centres at perovskite surfaces through O, N or S coordination sites, which may contribute to interfacial defect passivation and buried-interface stabilization [48]. Overall, substituent and interfacial functional-group engineering has expanded SAM design beyond early work-function tuning towards the integrated modulation of molecular polarity, interfacial dipoles, surface energy, defect binding and compatibility with subsequent perovskite deposition. This evolution provides a molecular basis for constructing multifunctional buried interfaces with coupled energetic, chemical and morphological regulation [36,48].

### 2.3. Emerging Conjugated-Backbone and Molecular-Orientation Design

Although PACz-based SAMs have become representative hole-selective contacts in high-efficiency inverted PSCs, the poor wettability of hydrophobic Me-4PACz surfaces remains an important processing limitation [49]. π-expanded carbazole backbones provide a representative route for broadening molecular geometry and electronic tunability beyond conventional PACz molecules [50]. More broadly, conjugated-backbone engineering aims to tune molecular energetics, conformational flexibility, packing behaviour and interfacial compatibility [36].

**Figure 4:**
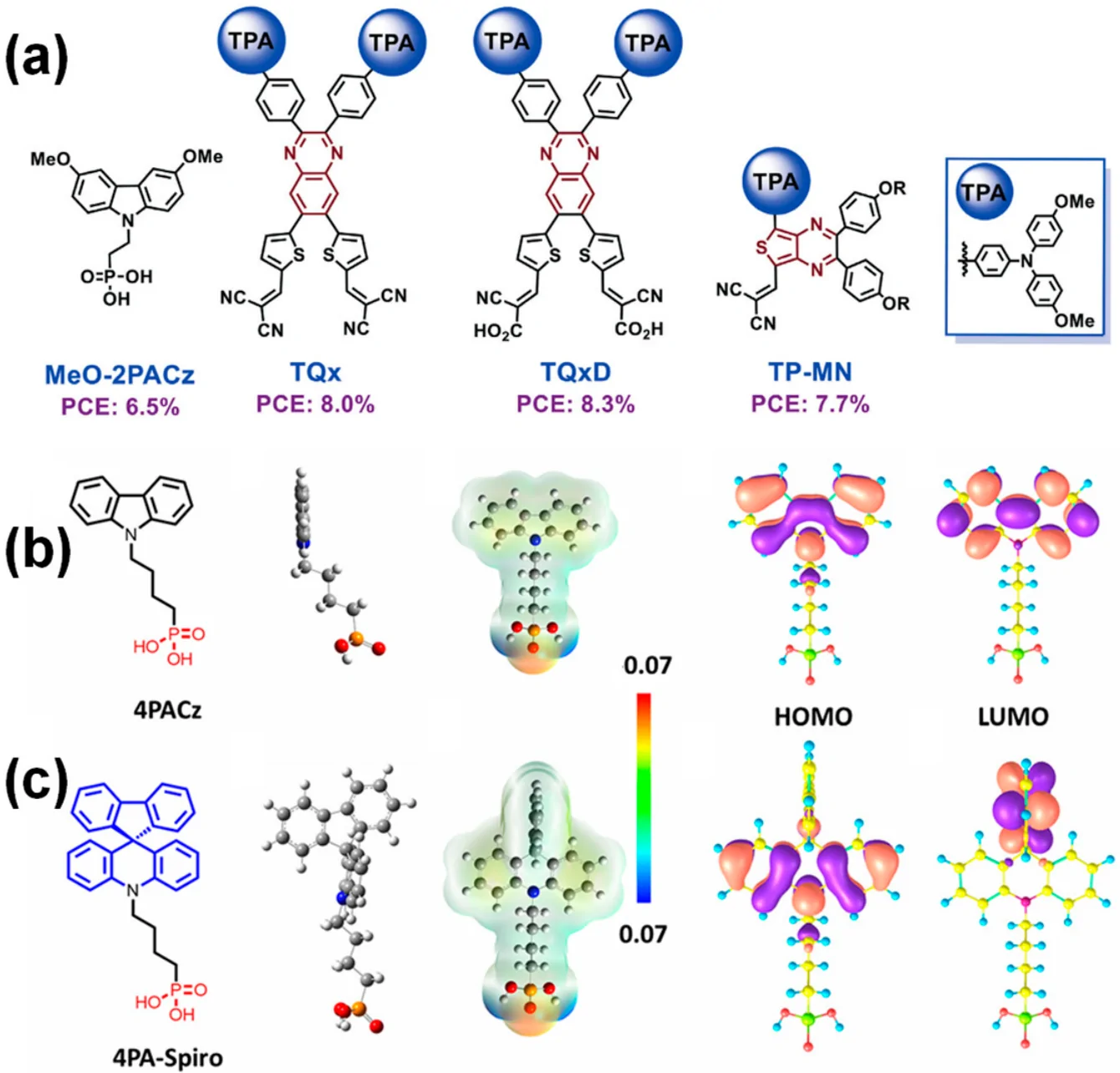
Emerging conjugated-backbone design of SAMs for inverted perovskite solar cells. (**a**) Chemical structures of MeO-2PACz and TPA-based SAMs, including TQx, TQxD and TP-MN Reprinted with permission from Ref. [51]. Copyright 2024 John Wiley and Sons. (**b**,**c**) Molecular structures, optimized configurations, electrostatic potential distributions and frontier molecular orbital distributions of 4PACz and 4PA-Spiro. Reprinted from Ref. [32].

Among non-PACz molecular backbones, triphenylamine (TPA) is attractive because of its electron-donating character and favourable hole-transport properties. Triphenylamine-based Y-shaped SAMs provide a representative example of using this backbone as a hole-selective contact in tin perovskite solar cells [51] (Figure 4a). Separately, a triphenylamine-based SAM containing dual coordination sites has been used to combine hole selection with buried-interface defect passivation [48]. Phenothiazine represents another attractive electron-rich conjugated backbone because of its strong electron-donating character, structural tunability and hole-transport capability. Recent studies have incorporated phenothiazine cores into SAM-forming molecules bearing oxide-binding anchoring groups, thereby enabling their use as hole-selective contacts in both wide-bandgap lead-based and tin-based perovskite solar cells [52,53]. These results demonstrate that phenothiazine provides a viable backbone platform for expanding SAM design beyond conventional PACz derivatives. A recent study reported a spiro-type SAM incorporating a spiro[acridine-9,9′-fluorene] core, highlighting steric-geometry engineering as a potential strategy for improving SAM coverage uniformity and device performance [32] (Figure 4b,c). Collectively, these studies indicate that SAM backbone design is evolving from the conventional PACz framework towards more diverse π-conjugated architectures, including TPA, phenothiazine and spirocyclic motifs.

Together, these examples demonstrate that conjugated-backbone engineering expands the molecular geometries, electronic structures and interfacial interactions available beyond the PACz family, providing additional opportunities to optimize coverage uniformity, energetic alignment and buried-interface compatibility. In addition to backbone expansion, molecular-orientation engineering has emerged as another useful strategy for optimizing SAM-based contacts, as exemplified by tripodal π-conjugated cores that promote face-on-oriented hole-collecting monolayers [54]. These advances indicate that SAM design is moving beyond PACz-based hole-selective contacts towards a broader molecular-design paradigm that integrates anchoring chemistry, substituent and interfacial functional-group engineering, conjugated-backbone diversification and molecular-orientation control. Together, these molecular design principles provide the structural basis for understanding how SAMs regulate buried interfaces through energy-level alignment, carrier extraction, defect passivation, crystallization control and stability enhancement, as summarized below and further discussed in Section 3 [20,27,36].

### 2.4. Summary of SAM Molecular Design Principles

Overall, SAM molecular design has evolved from benchmark PACz molecules towards substituent engineering, conjugated-backbone diversification and molecular-orientation control. PACz derivatives provide a relatively mature and structurally tunable platform for regulating interfacial energetics and surface properties; whereas, emerging molecular backbones expand the tunability of molecular dipoles, packing configurations and interfacial interactions. Nevertheless, the performance of a given SAM molecule remains strongly dependent on substrate chemistry, molecular coverage and deposition conditions, and improvement in one molecular descriptor may introduce trade-offs in wettability, packing behaviour or processing tolerance. Therefore, future molecular design should move beyond isolated energy-level optimization towards experimentally validated multiparameter optimization of molecular structure, assembly behaviour and device-relevant interfacial properties.

## 3. Mechanisms of SAM-Mediated Buried-Interface Engineering

SAM-mediated buried-interface engineering in inverted PSCs is governed by multiple coupled effects rather than by a single interfacial factor. Through interfacial dipoles, chemical interactions, surface-energy modulation and interfacial stabilization, SAMs can influence device performance by regulating energy-level alignment, selective carrier extraction, defect passivation, perovskite crystallization and long-term interface stability [20,23,55]. At the molecular level, SAM dipoles and electronic structures largely govern their ability to modulate the effective work function of the underlying electrode. Anchoring groups and terminal functionalities mediate interactions with oxide substrates and perovskite surfaces; whereas, molecular coverage, orientation and packing strongly influence the spatial uniformity and long-term stability of the buried interface [56].

### 3.1. Interfacial-Dipole Engineering for Energy-Level Alignment

Energy-level alignment is a key requirement for SAMs to function as efficient hole-selective contacts. When SAM molecules adsorb onto oxide surfaces such as ITO, FTO or NiO_x_ with preferential orientations, they can generate interfacial dipoles that shift the vacuum level and tune the effective work function of the underlying electrode [18,20]. In inverted PSCs, deepening the effective work function of the transparent conducting oxide toward the perovskite valence-band maximum can reduce hole-extraction barriers, strengthen hole selectivity and mitigate interfacial energetic losses [57]. Al-Ashouri et al. systematically explored carbazole phosphonic acid SAMs (2PACz, MeO-2PACz and Me-4PACz), as approximately 1 nm-thick ultrathin hole-selective contacts for inverted PSCs. These molecular layers enabled well-matched buried-interface energetic alignment and charge selectivity, highlighting the importance of interfacial-dipole engineering in SAM-based contacts [18]. Stolterfoht et al. [11] highlighted the close relationship between contact-interface energy-level alignment, interfacial non-radiative recombination and open-circuit voltage losses. This finding underscores the importance of tuning the underlying electrode work function through SAM-induced dipoles to achieve better energy-level alignment and low-loss hole-selective contacts.

Importantly, the interfacial energetics of SAM-modified contacts cannot be understood solely from the HOMO level of isolated molecules. Instead, they are shaped by a combination of molecular dipole moments, adsorption geometries, orientation angles, surface coverage and the chemical state of the oxide surface. Consistent with this view, Kralj and co-workers showed that the interfacial electronic properties of carbazole-based SAMs depend not only on molecular structure, but also on the microstructure of the TCO substrate. Surface morphology and local structural variations in the substrate can therefore contribute to spatial heterogeneity in work-function modulation [21]. These findings suggest that energy-level alignment in SAM-modified contacts should be viewed not simply as a molecular property, but as the result of coupled effects among molecular structure, interfacial dipoles and substrate surface states.

### 3.2. Selective Carrier Extraction and Suppression of Interfacial Non-Radiative Recombination

**Figure 5:**
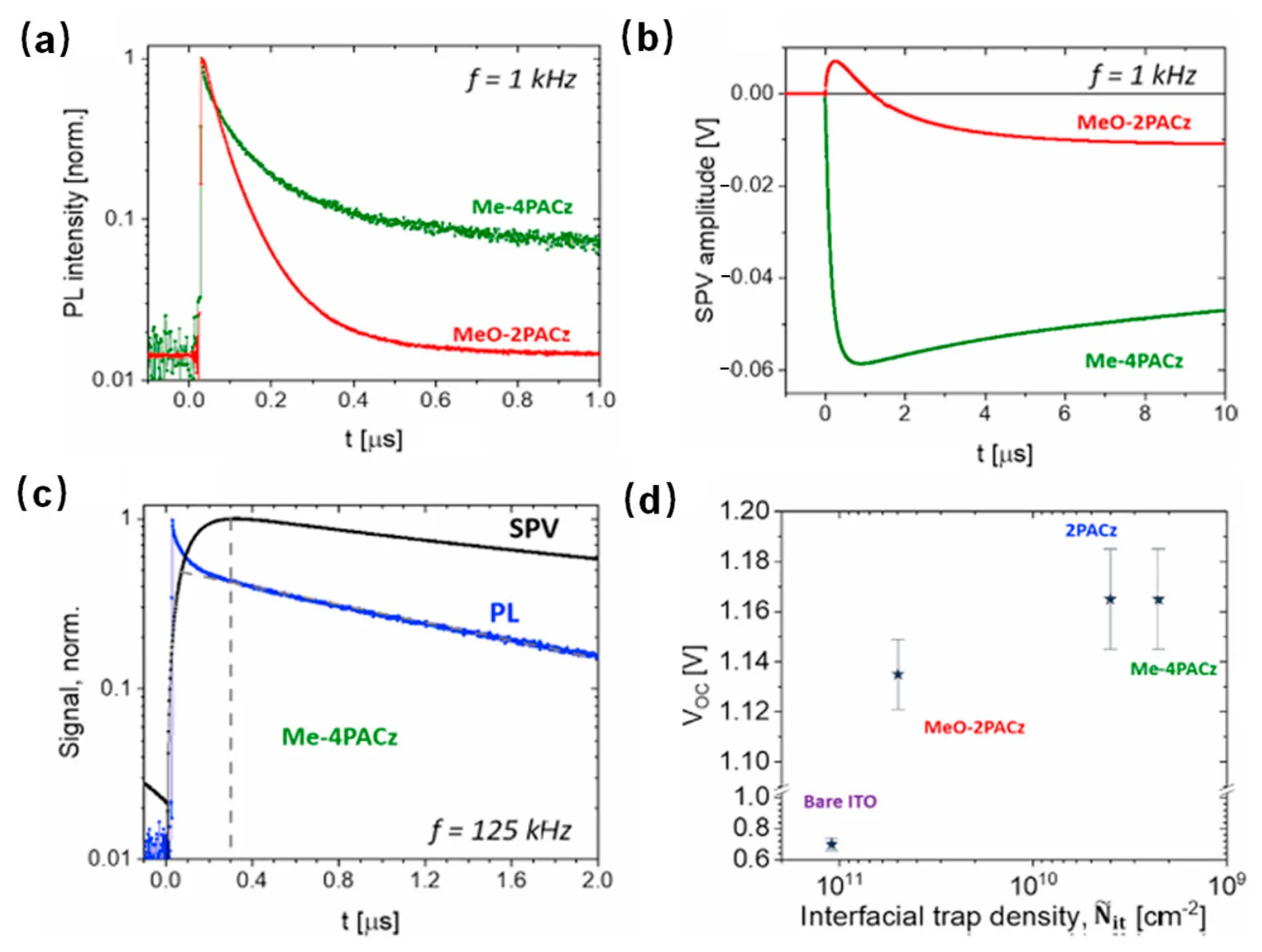
Carrier-transfer dynamics and interfacial trap-state analysis at SAM-modified ITO/perovskite buried interfaces. (**a**,**b**) tr-PL decay and tr-SPV responses of perovskite films on MeO-2PACz- and Me-4PACz-modified ITO substrates, (**c**) normalized PL and SPV transients for the Me-4PACz-modified interface, indicating efficient hole-transfer kinetics, (**d**) relationship between Voc and interfacial trap density for bare ITO and carbazole-based SAM-modified interfaces, highlighting the role of SAMs in reducing interfacial traps and suppressing recombination losses. Reprinted from Ref. [58].

Beyond static energy-level alignment, SAMs regulate carrier-transfer kinetics at buried interfaces. Efficient SAM contacts should promote rapid hole extraction from the perovskite absorber to the bottom electrode while suppressing electron leakage towards the hole-selective contact, thereby lowering the likelihood of interfacial charge recombination [58]. Using time-resolved surface photovoltage and time-resolved photoluminescence measurements, Levine and co-workers examined ITO/perovskite buried interfaces modified with different carbazole-based SAMs [58]. The Me-4PACz-modified interface showed a lower interfacial trap density and more favourable hole-transfer dynamics, indicating that SAMs influence not only interfacial energy-level alignment but also carrier extraction and trap-assisted recombination (Figure 5). Interfacial non-radiative recombination is often associated with energy-level mismatch, interfacial trap states and undesired carrier accumulation at contact interfaces. Uniform SAM coverage and appropriate energy-level alignment can mitigate bottom-electrode-induced local barriers and electron-trapping centres, thereby suppressing Shockley–Read–Hall (SRH) trap-assisted recombination [21,59]. The suppression of interfacial recombination losses helps increase the quasi-Fermi-level splitting (QFLS), thereby contributing to improved open-circuit voltage and fill factor [9].

### 3.3. Chemical Interactions for Interfacial Defect Passivation

**Figure 6:**
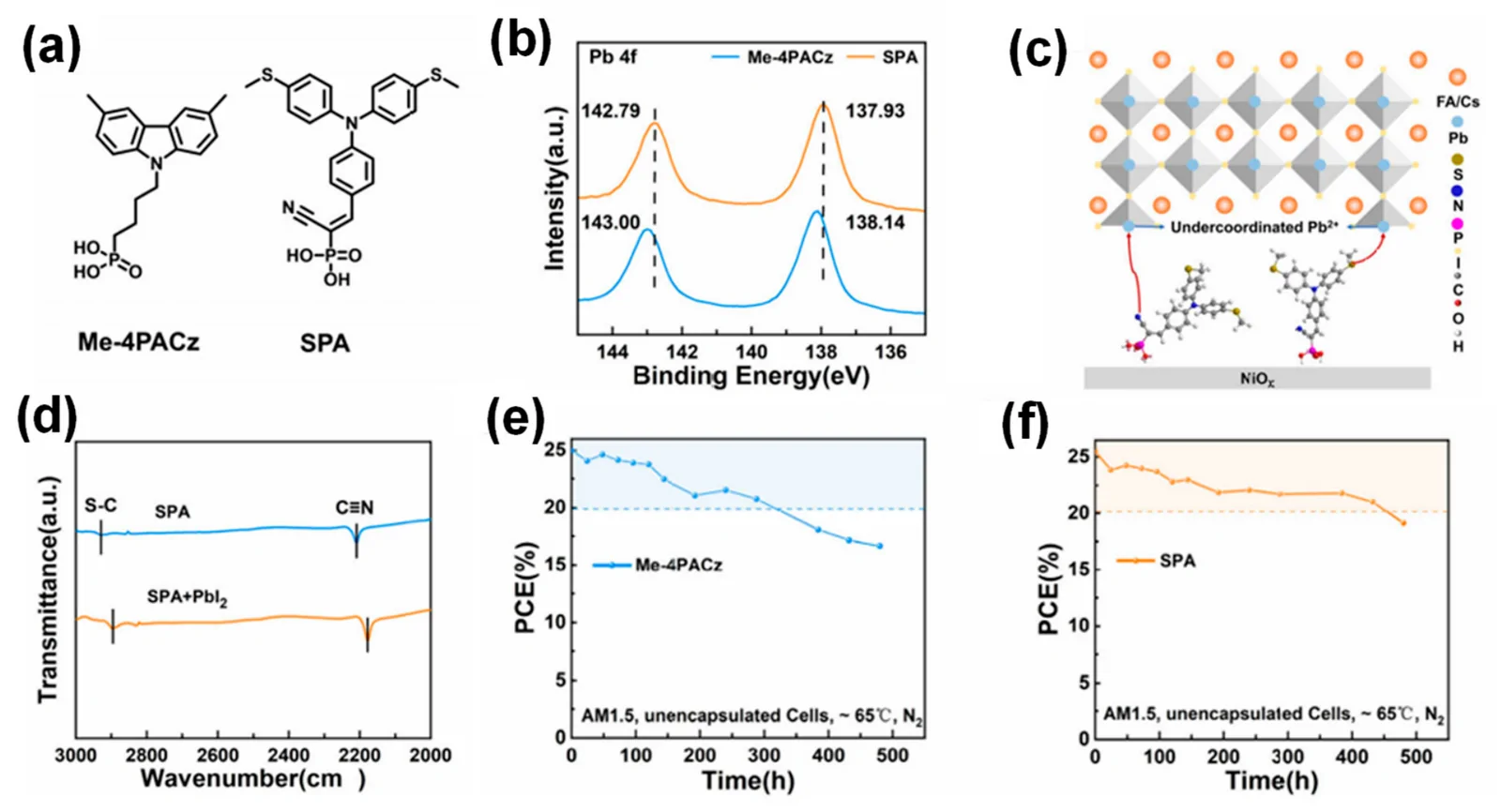
Molecular design, interfacial interaction, and device stability of the triphenylamine-based dual-site coordination SAM. (**a**) Molecular structures of Me-4PACz and SPA, (**b**) Pb 4f XPS spectra of perovskite films modified with different SAMs, (**c**) schematic illustration of the dual-site coordination interaction between SPA and the perovskite buried interface, (**d**) FTIR spectra of different SAM/perovskite samples. (**e**) Evolution of device efficiency based on Me-4PACz and SPA during ageing, (**f**) maximum power point tracking stability of the SPA-based device. Reprinted with permission from Ref. [48]. Copyright 2026 American Chemical Society.

Beyond their roles in energy-level alignment and carrier extraction, SAMs can passivate buried-interface defects through specific chemical interactions. Under-coordinated Pb^2+^ centres, halide vacancies and oxide-substrate-induced defects are commonly present at the bottom surface of perovskite films, where they can act as deep-level traps and promote non-radiative recombination [60]. Functional groups in SAM molecules, such as methoxy, carbonyl and cyano groups, as well as O-, N- or S-containing heteroatom motifs, can act as Lewis-basic or coordination sites. These sites may coordinate with under-coordinated Pb^2+^ centres or interact with other defect sites at the perovskite surface, thereby reducing the interfacial defect density and improving the chemical stability of the buried interface [48,59]. Yuan and co-workers reported a triphenylamine-based SAM with dual coordination sites, which interacted with the perovskite surface through molecular coordination sites and enabled interfacial defect passivation and improved stability [48] (Figure 6). Jiang et al. [59]. developed asymmetric SAM molecules, CbzBF and CbzBT, that incorporate Lewis-basic O and S heteroatom sites. In these molecules, interfacial dipoles tune the ITO work function; whereas, the heteroatom sites interact with defects at the bottom perovskite surface, enabling simultaneous hole-selective contact formation and buried-interface passivation. As a result, CbzBT-based devices achieved a PCE of 24.0% and a fill factor of 84.41%, together with improved stability.

SAM layers can further suppress interfacial recombination by screening chemically reactive oxide surfaces. Hydroxyl groups, oxygen vacancies and under-coordinated metal sites are often present on ITO, FTO or NiO_x_ surfaces, where they may promote defect formation at the bottom perovskite interface [22,61]. Through anchoring-group binding to oxide surfaces, SAMs can partially screen or passivate reactive surface sites and reduce undesired chemical contact between the perovskite absorber and the bottom electrode [20,22]. Phung et al. [22] further showed that ALD-grown NiO_x_ can promote more uniform SAM coverage, thereby improving interfacial uniformity and contact quality in inverted PSCs. Overall, the defect-passivation function of SAMs arises from two coupled interfacial processes: coordination with defect sites on the perovskite side and screening of reactive sites on the oxide-substrate side.

### 3.4. Surface-Energy Engineering for Perovskite Crystallization Control

**Figure 7:**
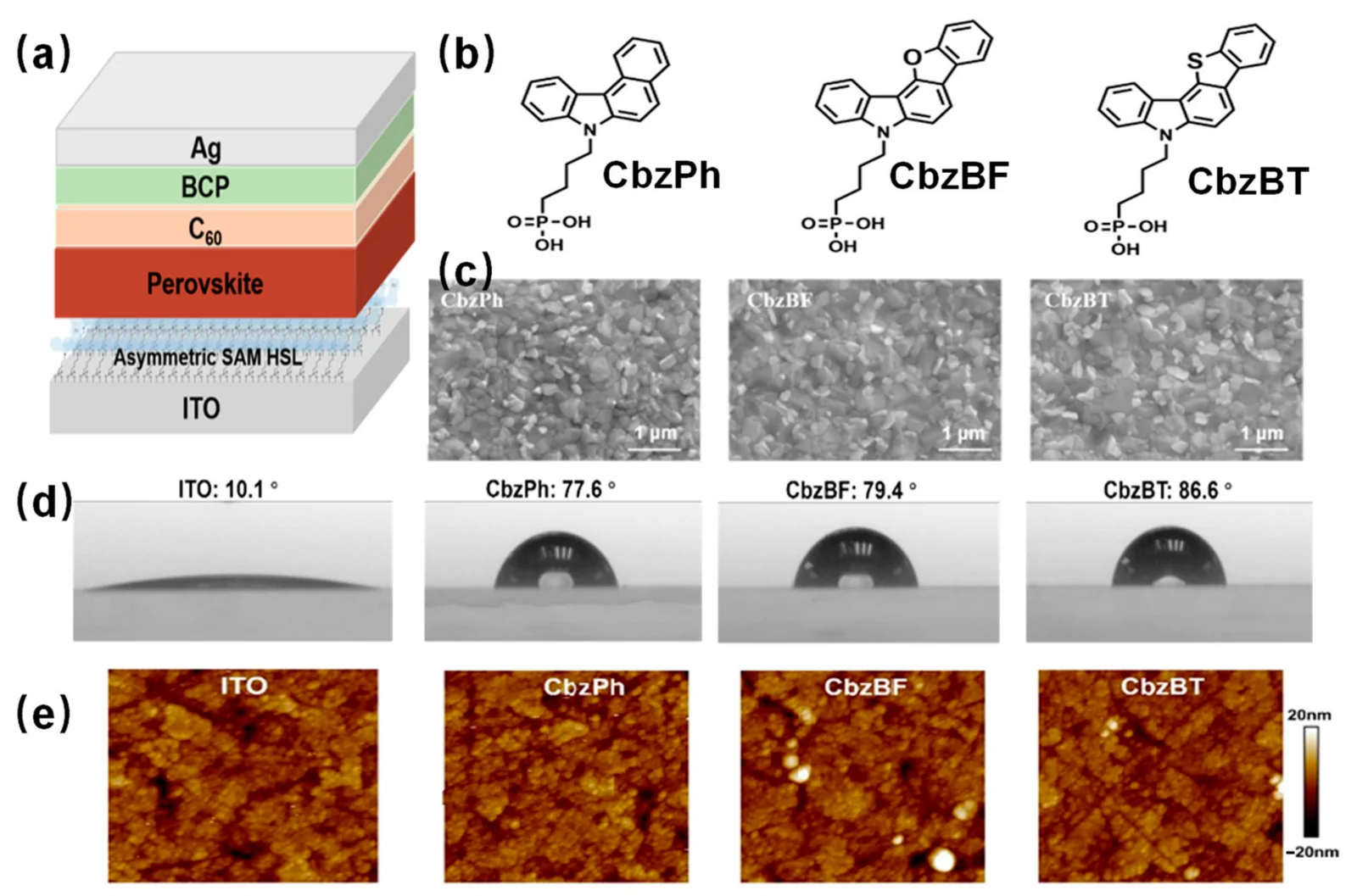
Surface-energy regulation by asymmetric SAMs. (**a**) Device architecture of inverted perovskite solar cells using an asymmetric SAM as the hole-selective layer. (**b**) Molecular structures of CbzPh, CbzBF and CbzBT. (**c**) Top-view SEM images of perovskite films deposited on different SAM-modified substrates. Scale bars: 1 μm. (**d**) Water-contact-angle images of ITO, CbzPh, CbzBF and CbzBT substrates. (**e**) AFM height images of ITO and SAM-modified substrates. Reprinted from Ref. [59].

As ultrathin interfacial layers between the perovskite absorber and the bottom electrode, SAMs shape the chemical environment of the underlying surface on which the perovskite precursor is deposited. Through terminal-group-dependent polarity and surface energy, SAMs can regulate precursor wetting and spreading, thereby influencing heterogeneous nucleation and film continuity [62] (Figure 7). For example, Jiang et al. [59] developed asymmetric SAM molecules, including CbzPh, CbzBF and CbzBT, and showed that heteroatom-containing molecular structures can regulate substrate wettability and surface morphology; the corresponding contact-angle, AFM and SEM results further indicate that SAM-dependent surface properties affect perovskite film formation at the buried interface. And Me-4PACz provides a representative example of this trade-off: although its hydrophobic surface can enable low-loss hole-selective contacts, it may compromise precursor wetting, leading to non-uniform local spreading and reduced reproducibility in perovskite film formation [49]. Kulkarni et al. [62] addressed this issue by modulating precursor–substrate interactions, which improved precursor wetting and uniform spreading on hydrophobic SAM surfaces and thereby enhanced the reproducibility of perovskite film formation. Al-Ashouri et al. [63] further highlighted the importance of wettability control in carbazole-based hole-selective SAMs. Improved surface wettability facilitates uniform precursor spreading, suppresses local dewetting and film discontinuity, and enhances the reproducibility of perovskite film formation.

Mechanistically, SAMs regulate perovskite crystallization by coupling surface-energy effects with interfacial chemical interactions and spatial heterogeneity. Surface energy governs precursor wetting, spreading and solvent-evaporation dynamics; terminal-group interactions with Pb^2+^, organic cations or halide centres modify the adsorption configuration of precursor centres at the interface; and SAM coverage uniformity and local potential distribution determine the spatial distribution of heterogeneous nucleation sites [60,62].Taken together, wettability, surface energy and interfacial chemical interactions should be considered as coupled parameters in SAM design. Their rational control can promote uniform perovskite film formation while mitigating interfacial recombination losses arising from film non-uniformity and buried-interface defects.

### 3.5. Sam-Mediated Interfacial Stabilization and Ion-Migration Suppression

Long-term buried-interface stability is essential for achieving both high efficiency and durable operation in inverted PSCs [64]. For SAM-based devices, this requires SAM layers that not only ensure desirable energy-level alignment and hole-selective contact formation, but also preserve robust interfacial binding and molecular coverage under thermal, light and moisture stress [40]. SAMs can enhance buried-interface stability through two related mechanisms. Anchoring groups strengthen the binding between the bottom electrode and the molecular layer and partially screen reactive oxide-surface sites, which helps reduce defect-induced non-radiative recombination and interfacial degradation [20,22]. Meanwhile, SAMs with high coverage integrity and robust binding can suppress ion accumulation at buried interfaces and limit detrimental interfacial reactions, thereby mitigating performance degradation caused by ion migration, local charge build-up and interfacial chemical evolution [55,65]. Beyond SAM-specific systems, the importance of molecular reactivity has also been highlighted by ligand-engineering studies, where rationally designed passivators can improve high-temperature operation by providing interfacial passivation while minimizing detrimental reactions with the perovskite lattice [66]. Because these stability results were obtained under different encapsulation, illumination, temperature, atmosphere and tracking conditions, they should be regarded as representative examples rather than directly comparable benchmarks. The stability of SAM-based interfaces is difficult to compare quantitatively across different studies because the reported results depend strongly on the testing protocol. Important variables include device encapsulation, illumination spectrum and intensity, operating temperature, humidity, atmosphere, electrical bias, maximum-power-point tracking conditions, device area and the definition of the stability endpoint [67]. Moreover, the measured device degradation may originate not only from the SAM contact, but also from perovskite bulk decomposition, ion migration, top-interface reactions, electrode corrosion or encapsulation failure [68]. Therefore, stability data should be interpreted together with the complete device architecture and testing conditions, and direct comparisons should preferably be limited to devices evaluated under consistent or standardized protocols [67]. Liu et al. [23]. developed a co-assembled buried-interface layer by combining Me-4PACz with the multi-aromatic carboxylic acid 4,4′,4″-nitrilotribenzoic acid (NA). The resulting molecular hybrid layer improved SAM/perovskite interfacial contact, reduced interfacial losses and enhanced operational stability. This strategy enabled inverted PSCs with a certified stabilized PCE of 26.54%, as well as 11.1 cm^2^ mini-modules with an aperture-area efficiency of 22.74% and 96.1% retention of the initial efficiency after 2400 h of continuous operation. Consistent with this picture, Yu et al. [55] investigated buried-interface stabilization strategies in SAM-based inverted devices and highlighted the close coupling among molecular anchoring, interfacial defects and ion-migration processes in determining interfacial stability. Jiang et al. [34]. introduced a toughened-SAM strategy to enhance the thermal robustness of the buried interface. The stabilized SAM layer reduced substrate exposure and perovskite decomposition associated with the thermal motion of loosely bound SAM molecules, thereby improving device stability under thermal stress. This approach enabled devices with a certified PCE of 26.92%, negligible degradation after 1000 h of maximum-power-point tracking at 85 °C, and over 98% retention of the initial efficiency after 700 thermal cycles between −40 and 85 °C.

However, conventional small-molecule SAMs do not always form ideal, well-defined monolayers under practical processing conditions. Their assembly is sensitive to substrate hydroxyl density, surface roughness, weakly adsorbed molecules and subsequent solvent exposure, which can generate non-uniform coverage, local substrate exposure and molecular desorption. These complexities indicate that real SAM interfaces should be viewed as structurally heterogeneous interfacial layers, whose binding motifs, layer distribution and stability strongly affect subsequent perovskite deposition and device performance [69]. Potential negative effects associated with SAM-modified interfaces should also be considered during long-term operation. First, polar solvents used during perovskite precursor deposition may partially remove weakly adsorbed or multilayer SAM molecules, leading to local substrate exposure, interfacial-dipole fluctuation and reduced contact selectivity [33,69]. Second, thermal stress and illumination can promote molecular rearrangement or the motion of loosely bound SAM molecules, which may change molecular orientation, surface coverage and local work-function distribution at the buried interface [34,40]. Third, environmental ageing under moisture, oxygen and thermal stress may weaken molecule–substrate binding or accelerate interfacial chemical reactions, especially at regions with incomplete SAM coverage or reactive oxide-surface sites [40,70,71]. These possible degradation pathways indicate that SAM stability should be evaluated not only by initial device efficiency, but also by solvent resistance, anchoring robustness, coverage retention and interfacial chemical stability under realistic processing and operating conditions.

### 3.6. Summary of SAM-Mediated Buried-Interface Mechanisms

Interfacial dipoles tune the effective work function of the bottom electrode and help reduce hole-extraction barriers [20], while chemical interactions between SAM functional groups and interfacial defect sites suppress trap-assisted non-radiative recombination [60]. In parallel, surface energy and wettability determine precursor spreading, heterogeneous nucleation and subsequent grain growth. The stability of the buried interface is further affected by anchoring strength, molecular coverage and resistance to solvent exposure, thermal stress and ion accumulation.

**Figure 8:**
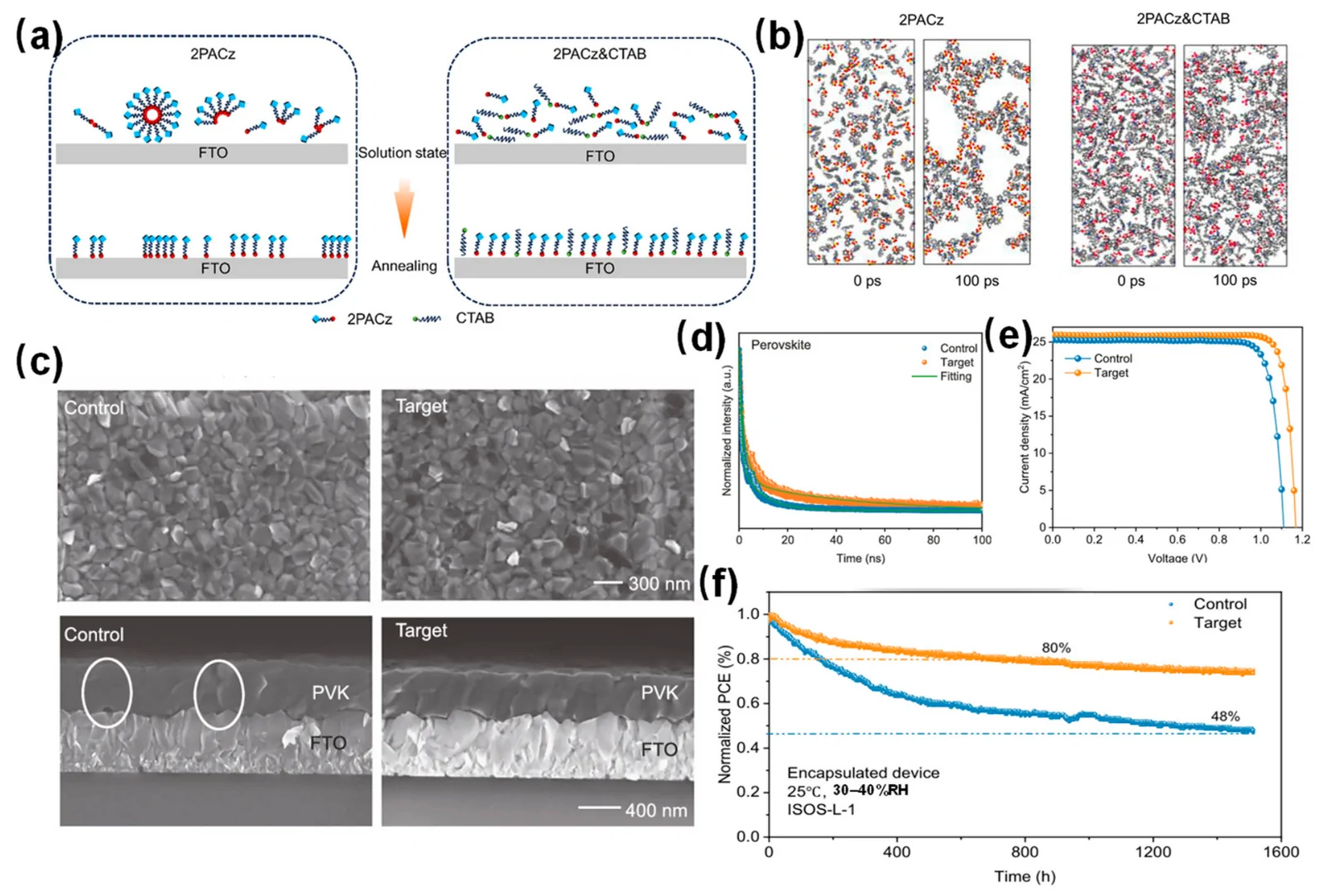
(**a**) Schematic illustration of CTAB-assisted regulation of 2PACz aggregation and SAM formation on FTO. (**b**) Molecular dynamics simulation of 2PACz and 2PACz&CTAB during the SAM formation process. (**c**) Top-view and cross-sectional SEM images of perovskite films deposited on control and target SAM-modified substrates. (**d**) TRPL spectra of perovskite films based on control and target SAMs. (**e**) J–V curves of control and target devices. (**f**) Operational stability of encapsulated devices under ISOS-L-1 conditions. Reprint from Ref. [72].

A representative example linking these processes is shown in Figure 8. In this case, the regulation of 2PACz aggregation on the FTO substrate affects SAM coverage and molecular distribution. Improved SAM coverage is associated with more uniform perovskite film formation, enlarged grains, reduced grain-boundary density and slower carrier recombination [72]. These changes are further reflected in enhanced device efficiency and improved operational stability. This example illustrates the relationship among SAM formation on practical oxide substrates, perovskite nucleation and growth, and device performance.

Taken together, the buried-interface function of SAMs should be understood as the combined result of molecular structure, substrate surface state, assembly behaviour and subsequent perovskite deposition. Therefore, the design of high-performance SAMs requires simultaneous consideration of adsorption strength, molecular orientation, surface coverage, precursor wettability, defect coordination and interfacial stability. The molecular design principles discussed in Section 2 provide the structural basis for these functions, while the emerging strategies discussed in Section 4 further extend this mechanism-oriented design towards more integrated SAM-based buried interfaces.

## 4. Emerging Strategies for Sam-Based Interface Engineering

With the continued improvement in inverted PSC efficiency, SAMs are evolving beyond conventional ultrathin hole-selective contacts into multifunctional buried-interface engineering platforms. In this role, they integrate energy-level modulation, interfacial defect passivation, selective carrier extraction and stability enhancement within a molecularly thin interfacial layer [19,35,73]. Nevertheless, it remains challenging for conventional single-component SAMs to reconcile energy-level alignment, surface wettability, interfacial coverage and long-term stability within one molecular system [49]. This trade-off is exemplified by Me-4PACz, which provides efficient hole-selective contact formation but often suffers from limited wettability towards perovskite precursor inks, leading to reduced film uniformity and device reproducibility [62,63]. Beyond intrinsic molecular trade-offs, SAM formation on electrode surfaces is highly sensitive to substrate hydroxyl density, anchoring chemistry and deposition protocols, which determine adsorption density, molecular orientation and coverage uniformity. Local non-uniformity in SAM deposition therefore remains a key challenge for scaling SAM-based buried-interface engineering [61]. These challenges have driven SAM research in recent years to move beyond single-molecule optimization towards multidimensional interface-engineering strategies, including Co-SAMs, amorphous SAMs and polymerized SAMs [31,33,74].

### 4.1. Co-Assembled Sams

Co-SAM strategies are designed to overcome the intrinsic limitations of single-component SAMs by using multicomponent molecular co-assembly to achieve multifunctional buried-interface regulation. In this approach, a second molecular component is introduced so that different molecules can contribute complementary functions, including energy-level modulation, improved wettability, defect passivation and interfacial stabilization [29]. Through co-adsorption or sequential deposition, these functions can be integrated within one buried interface [75,76,77]. Accordingly, Co-SAMs are not simply physical mixtures of two molecular species. Instead, they use multicomponent interfacial synergy to expand the design space for simultaneously regulating energy-level alignment, precursor wetting, molecular packing and operational stability [78].

**Figure 9:**
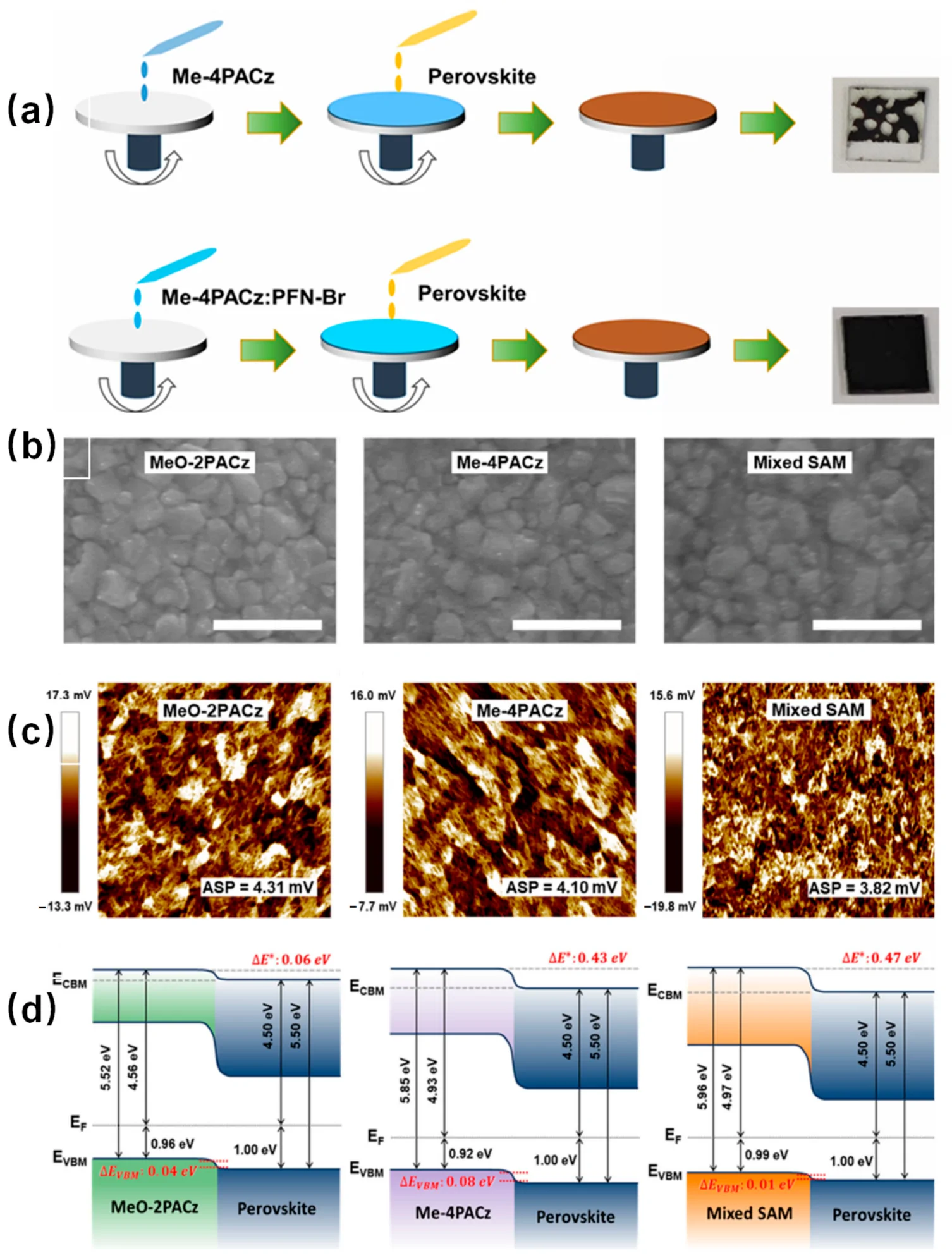
Regulation of perovskite film formation and interfacial energy-level alignment by composite interfaces and mixed SAMs. (**a**) Schematic illustration of perovskite deposition on Me-4PACz and Me-4PACz:PFN-Br substrates, together with photographs of the resulting perovskite films, showing improved film coverage after PFN-Br incorporation Reprinted with permission from Ref. [49]. Copyright 2023 American Chemical Society. (**b**) SEM images of perovskite films deposited on MeO-2PACz, Me-4PACz, and MeO-2PACz/Me-4PACz mixed SAM substrates, (**c**) SKPM surface-potential maps of perovskite films on MeO-2PACz, Me-4PACz, and mixed SAM substrates, and (**d**) schematic energy-level alignment at the MeO-2PACz/perovskite, Me-4PACz/perovskite, and mixed SAM/perovskite interfaces. ΔE* represents the energy-level offset between the SAM and perovskite considering Fermi-level alignment. Reprinted with permission from Ref. [77]. Copyright 2026 John Wiley and Sons.

One important advantage of Co-SAMs is their ability to broaden the tuning window for interfacial dipoles and energy-level alignment. Because different SAM molecules have distinct dipole moments, molecular tilt angles and electronic structures, their co-assembly can generate coupled dipole effects that enable more precise modulation of the bottom-electrode work function. This provides a route to more desirable energetic alignment between the bottom electrode and the perovskite valence band. A representative example is the co-assembly of MeO-2PACz and Me-4PACz. MeO-2PACz provides relatively desirable wettability towards perovskite precursor inks, facilitating perovskite nucleation and film coverage; whereas, Me-4PACz offers a deeper HOMO level that improves interfacial energetic alignment and hole-selective transport. Their co-assembly therefore combines improved film formation with desirable energy-level alignment, enabling concurrent gains in device performance and stability [77] (Figure 9b–d). This example highlights a key feature of Co-SAMs: they enable a functional division of labour among different molecular components, followed by reintegration of these complementary functions within a co-assembled buried interface.

Co-SAMs provide an effective route to addressing film-formation problems caused by insufficient wettability in single-component SAMs. On highly hydrophobic PACz-based surfaces, perovskite precursor inks are prone to dewetting, leading to void formation, local thickness fluctuations and increased heterogeneity at the buried interface [49,62]. Hossain et al. [49]. reported a Me-4PACz/PFN-Br hybrid interface by combining Me-4PACz with the conjugated polyelectrolyte PFN-Br. The incorporation of PFN-Br improved the wettability of the otherwise hydrophobic Me-4PACz surface, leading to more uniform perovskite film coverage. PFN-Br also contributed to work-function tuning and interactions with A-site cations in the perovskite, which helped improve crystallization quality and device reproducibility (Figure 9a). These results indicate that Co-SAM and hybrid-interface strategies regulate film formation not merely by changing the surface contact angle, but also by strengthening precursor–substrate interactions and thereby modulating perovskite nucleation kinetics.

Co-SAMs can improve molecular packing and coverage uniformity at buried interfaces. For single-component SAMs, adsorption on TCO surfaces is highly sensitive to substrate hydroxyl density, molecular concentration, solvent environment and annealing conditions, often resulting in local undercoverage or molecular aggregation. The introduction of a second component provides an additional means of regulating lateral molecular packing through differences in steric effects, adsorption strength and molecular size, thereby filling interfacial gaps and mitigating spatial fluctuations in surface potential. He et al. [76] further demonstrated a precisely controlled Co-SAM strategy, highlighting the step-dependent functions of sequential molecular deposition during co-assembly. In this strategy, the initial deposition step primarily regulates surface wettability and film-formation uniformity; whereas, subsequent deposition steps further optimize energy-level alignment and hole extraction. These results suggest that the benefits of Co-SAMs are not determined solely by molecular composition, but also by the controllability of assembly sequence and interface-formation kinetics.

Co-SAMs can also mitigate the stability limitations associated with single-component SAMs. During long-term operation of inverted devices, mobile ions in the perovskite may migrate towards the SAM/ITO interface under electric fields and thermal stress, triggering interfacial chemical reactions, work-function drift and enhanced non-radiative recombination. The MeO-2PACz/Me-4PACz mixed SAM has also been shown to suppress MA^+^ migration towards the ITO side, helping improve operational stability under high-temperature operation [77]. This indicates that Co-SAMs are not merely efficiency-oriented optimization tools. They can also function as ion-migration barriers and interfacial-stabilization strategies.

**Figure 10:**
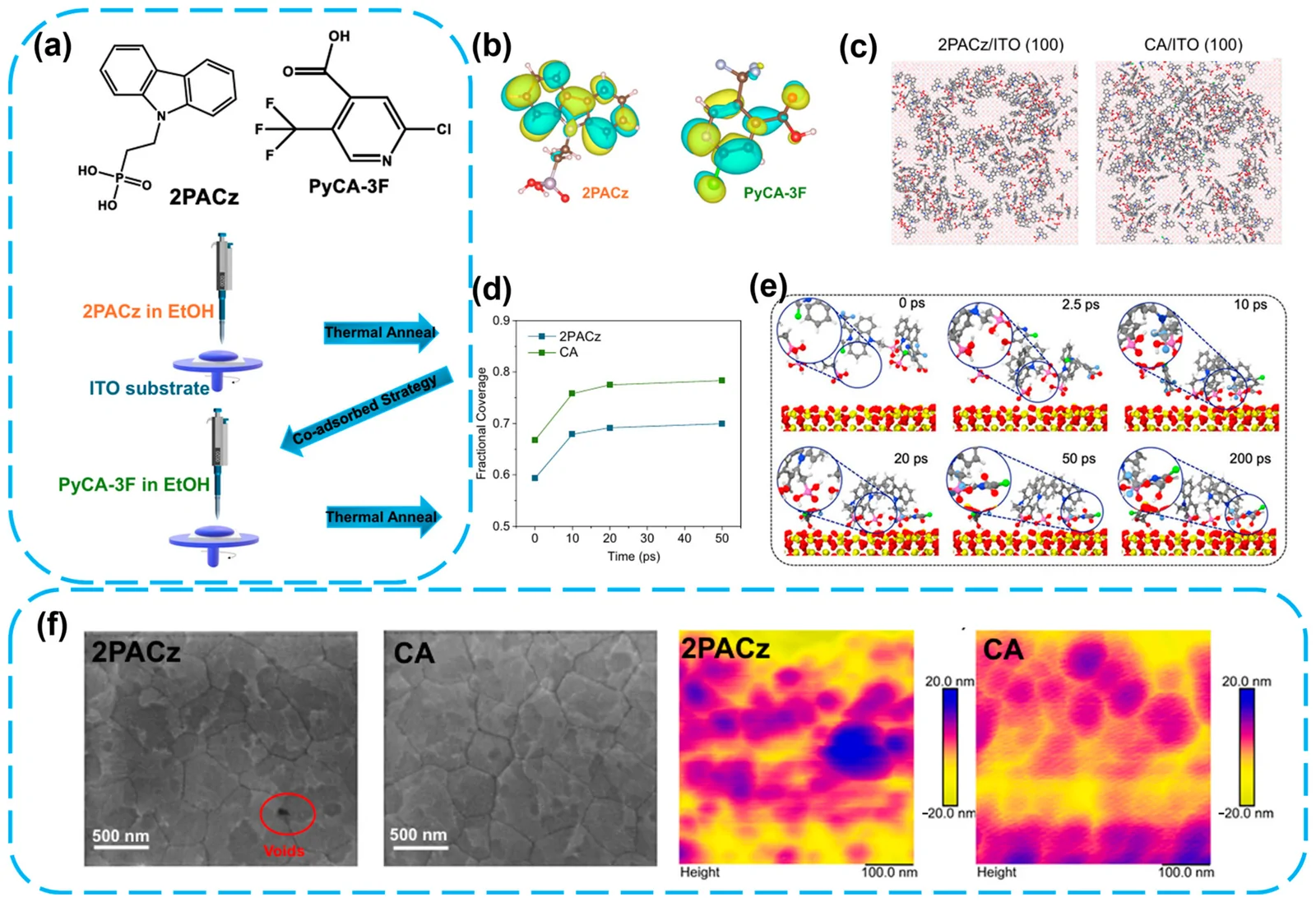
Construction of the 2PACz/PyCA-3F co-adsorbed SAM and its regulation of the perovskite buried interface. (**a**) Molecular structures of 2PACz and PyCA-3F, together with the schematic illustration of the co-adsorption process on the ITO substrate, (**b**) electrostatic potential distributions of 2PACz and PyCA-3F molecules, (**c**) simulated molecular coverage on 2PACz/ITO and CA/ITO surfaces, (**d**) time-dependent fractional coverage of 2PACz and CA on ITO. (**e**) Adsorption process and intermolecular interactions of 2PACz/PyCA-3F on the ITO surface, (**f**) SEM images and AFM height maps of perovskite buried interfaces formed on 2PACz and CA substrates. The CA co-adsorbed interface reduces interfacial voids observed on the 2PACz substrate and improves the uniformity of the perovskite buried interface. Reprinted from Ref. [75].

Li et al. [75]. demonstrated a co-adsorbed SAM interface based on 2PACz/2-chloro-5-(trifluoromethyl)isonicotinic acid (PyCA-3F). In this design, PyCA-3F, which bears a pyridine-carboxylic-acid motif and trifluoromethyl substituents, was introduced into the conventional 2PACz hole-selective contact and co-adsorbed with 2PACz on the bottom-electrode surface. This co-adsorption strategy enabled the formation of a Co-SAM layer for synergistic buried-interface regulation. In the 2PACz/PyCA-3F Co-SAM, 2PACz primarily serves as the hole-selective contact and tunes the electrode work function; whereas, PyCA-3F provides additional interfacial functions through its pyridine-carboxylic-acid motif and fluorinated groups. These structural motifs further modulate interfacial dipoles, strengthen interactions with the buried perovskite interface and reduce interfacial energetic losses (Figure 10). Notably, this strategy has also been extended to organic solar cells, suggesting that Co-SAM design may offer cross-system applicability beyond PSCs. Compared with single-component 2PACz, the key advance of 2PACz/PyCA-3F is the introduction of a second molecular component that supplements interfacial passivation and dipole modulation, thereby transforming the SAM interface from a simple hole-selective layer into a multifunctional buried-interface platform.

Overall, Co-SAMs address several key bottlenecks of single-component SAMs, including the limited tuning window of a single interfacial dipole, the poor wettability of efficient PACz-based SAMs, non-uniform molecular packing and coverage defects, and ion-migration-induced instability during long-term operation. In this sense, Co-SAMs mark an important shift in SAM-based interface engineering from single-molecule structural optimization towards multicomponent synergistic interface design. By coordinating the adsorption behaviour, interfacial dipoles, surface energy and packing modes of different molecular components, Co-SAMs provide a more flexible design space for balancing energy-level alignment, precursor wetting, interfacial coverage and operational stability [75,76,77]. Nevertheless, the multicomponent nature of Co-SAMs introduces additional challenges, including competitive adsorption, precise control of component ratios and lateral distribution uniformity. These factors may affect interfacial reproducibility during large-area coating and module fabrication, and therefore require further optimization [49,76].

### 4.2. Amorphous Sam

Amorphous SAMs have emerged as a strategy to overcome several limitations of conventional highly ordered SAMs at practical device interfaces. These limitations include local structural heterogeneity, non-uniform perovskite nucleation and insufficient long-term structural stability [21,31,35]. Conventional SAM design generally relies on ordered molecular orientation and dense packing to form well-defined interfacial dipoles and favourable charge-selective contacts. However, the bottom electrodes used in PSCs are far from ideal flat and chemically homogeneous surfaces. Oxide substrates such as ITO, FTO and NiO_x_ often contain surface roughness, grain boundaries, spatial variations in hydroxyl density and local chemical heterogeneity. As a result, an overreliance on long-range molecular ordering may make conventional SAMs vulnerable to local undercoverage, orientational disorder and surface-potential fluctuations on practical substrates [21]. Moreover, highly ordered molecular layers may introduce discrete nucleation sites for perovskite growth, resulting in spatial variations in grain size, crystallographic orientation and phase distribution. Strong intermolecular π–π stacking or crystallization tendencies within the SAM layer can also make the interface vulnerable to structural deformation or phase transitions under thermal, illumination and mechanical stress, thereby compromising long-term stability. In this context, amorphous molecular contacts enabled by orthogonal π-conjugated backbones offer a promising alternative, as they can better tolerate external stressors while preserving charge selectivity and transport capability [79]. Accordingly, amorphous SAMs are not intended to merely disrupt molecular ordering. Instead, they represent an amorphization-oriented molecular design strategy that aims to reduce interfacial structural heterogeneity while preserving essential interface functions such as charge selectivity, energetic alignment and interfacial stability [31,79].

**Figure 11:**
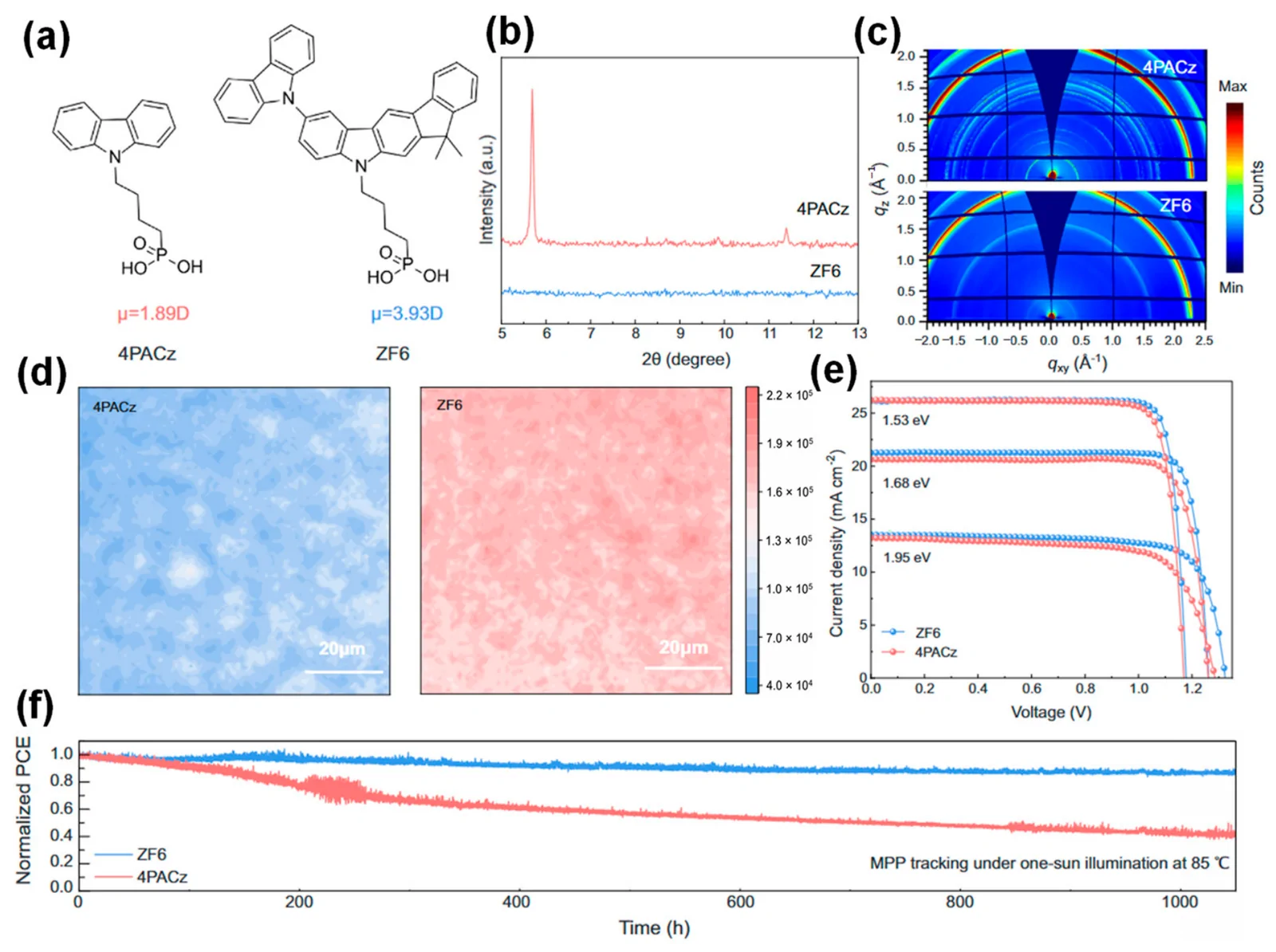
Amorphous and asymmetric molecular contact for efficient and stable inverted perovskite solar cells. (**a**) Molecular structures and dipole moments of 4PACz and ZF6. (**b**) XRD patterns and (**c**) GIWAXS images of 4PACz and ZF6 molecular layers, showing the amorphous characteristics of ZF6. (**d**) PL mapping images of perovskite films deposited on 4PACz- and ZF6-modified substrates. (**e**) J–V curves of ZF6- and 4PACz-based devices with different perovskite bandgaps. (**f**) Operational stability of ZF6- and 4PACz-based devices under one-sun illumination at 85 °C. Reprinted with permission from Ref. [80]. Copyright 2026 John Wiley and Sons.

As a representative amorphous-SAM strategy, [4-(3,6-diphenyl-9H-carbazol-9-yl)butyl] (PhPACz)-based molecular contacts move beyond conventional crystalline SAM architectures and help mitigate non-uniform perovskite growth as well as spatially uneven phase distribution at the buried interface [31,35]. Wang et al. [31]. compared crystalline Me-4PACz with amorphous PhPACz and showed that the rotatable phenyl groups in PhPACz disrupt regular intermolecular packing, thereby providing a more homogeneous nucleation interface for perovskite growth. Hyperspectral PL mapping revealed a narrower PL peak distribution for perovskite films grown on PhPACz, suggesting reduced local bandgap fluctuations and suppressed phase heterogeneity. Consistently, PhPACz-based films exhibited lower trap-assisted recombination, and 1 cm^2^ inverted devices achieved a PCE of 25.20% with improved light and thermal stability [31]. More recently, Chen et al. [80] developed an amorphous and asymmetric hole-selective molecular contact, ZF6, for efficient and stable perovskite/silicon tandem solar cells. As shown in Figure 11, the stronger molecular dipole and asymmetric π-extended structure of ZF6 promote amorphous molecular packing and improved perovskite-film uniformity, as verified by XRD, GIWAXS and PL mapping. ZF6-based tandem devices achieved a reverse-scan PCE of 34.02% and a certified PCE of 32.81%, while retaining over 97% and 87% of the initial efficiency after 1000 h of MPP tracking at 45 °C and 85 °C, respectively. Thus, amorphous SAMs do not simply abandon molecular ordering; instead, they use amorphization-oriented molecular design to reduce interfacial heterogeneity, improve film-formation uniformity and enhance long-term stability while preserving energy-level regulation, hole selectivity and interfacial passivation.

Orthogonal π-conjugated molecular contacts further highlight the value of amorphization-oriented design for improving the structural stability of buried interfaces. Zhou et al. [79] developed molecular contacts based on an orthogonal π-conjugated skeleton, in which the non-planar and orthogonal configuration disrupts the excessive π–π stacking commonly observed in planar conjugated backbones and induces amorphization of the molecular layer. The resulting spatially distorted amorphous contact layer improves interfacial tolerance to external stressors while preserving favourable charge selectivity and transport capability. These findings suggest that amorphous SAMs can enhance interfacial stability without sacrificing charge transport. Rather than simply introducing disorder, amorphization-oriented design weakens excessive molecular crystallization tendencies, thereby improving the long-term structural robustness of buried interfaces.

Spiro-type SAMs further extend this amorphization-oriented design concept by introducing non-planar molecular backbones that disrupt regular packing. Zhang et al. [32] developed (4-(10H-spiro[acridine-9,9′-fluoren]-10-yl)butyl)phosphonic acid (4PA-spiro), a spiro-type SAM molecule based on an orthogonal spiro[acridine-9,9′-fluorene] backbone. The twisted configuration and large steric hindrance of this backbone suppress intermolecular aggregation while preserving desirable energy-level alignment. Compared with conventional 4PACz, 4PA-spiro mitigates the local aggregation associated with planar molecular backbones and promotes a more uniform molecular distribution on the substrate, thereby reducing non-radiative recombination at the buried interface. As a result, 4PA-spiro-based devices achieved a PCE of 25.28% and a certified PCE of 24.81%, while 29.0 cm^2^ modules reached a PCE of 21.87%. These results suggest that non-planar and amorphization-oriented SAM design can enhance not only small-area device performance but also interfacial uniformity and scalability in large-area modules.

Taken together, amorphous and amorphization-oriented SAMs address several limitations of conventional ordered SAMs. PhPACz-based interfaces improve perovskite nucleation continuity and phase homogeneity; whereas, ZF6 further demonstrates that asymmetric π-extended molecular contacts can form amorphous interfacial layers with improved film uniformity and thermal operational stability. Orthogonal π-conjugated molecular contacts enhance the structural stability of SAM interfaces under thermal, illumination and mechanical stress by weakening excessive ordered packing; whereas, 4PA-spiro suppresses molecular aggregation through its twisted spiro backbone and improves interfacial uniformity during area scale-up. Thus, amorphous SAMs do not simply abandon molecular ordering; instead, they use amorphization-oriented molecular design to reduce interfacial heterogeneity, improve film-formation uniformity and enhance long-term stability while preserving energy-level regulation, hole selectivity and interfacial passivation.

### 4.3. Polymerized Sam

Polymerized and crosslinked SAMs have emerged as promising strategies to overcome the limitations of conventional small-molecule SAMs. Intermolecular crosslinking can improve coverage integrity, resistance to polar-solvent processing and long-term operational stability [33]. Polymeric backbone formation can further enhance film continuity, processing tolerance and compatibility with scalable coating [30]. Conventional small-molecule SAMs are typically only ~1–2 nm thick, and their interfacial integrity is maintained mainly by localized anchoring-group binding to the TCO surface and weak intermolecular interactions. Although this molecularly thin configuration can minimize parasitic absorption and facilitate low-loss carrier extraction when favourable energetic alignment is achieved [18], SAM formation remains sensitive to the surface chemistry of the oxide substrate. In particular, the surface hydroxyl density strongly affects phosphonic-acid anchoring and SAM coverage uniformity. Insufficient SAM coverage can leave regions of the ITO, FTO or NiO_x_ surface exposed and compromise interfacial contact quality [61]. Weakly adsorbed or multilayer SAM molecules may also be unstable during subsequent solution processing [69]. Polar solvents used for perovskite precursor deposition can induce molecular desorption and interfacial rearrangement [33]. Accordingly, intermolecular crosslinking can improve coverage continuity, resistance to polar-solvent processing and operational stability while retaining hole selectivity [33]. Polymeric SAM analogues can enhance film continuity and processing tolerance [30]; whereas, covalently linked bilayer structures can strengthen interfacial adhesion and thermal robustness [71].

**Figure 12:**
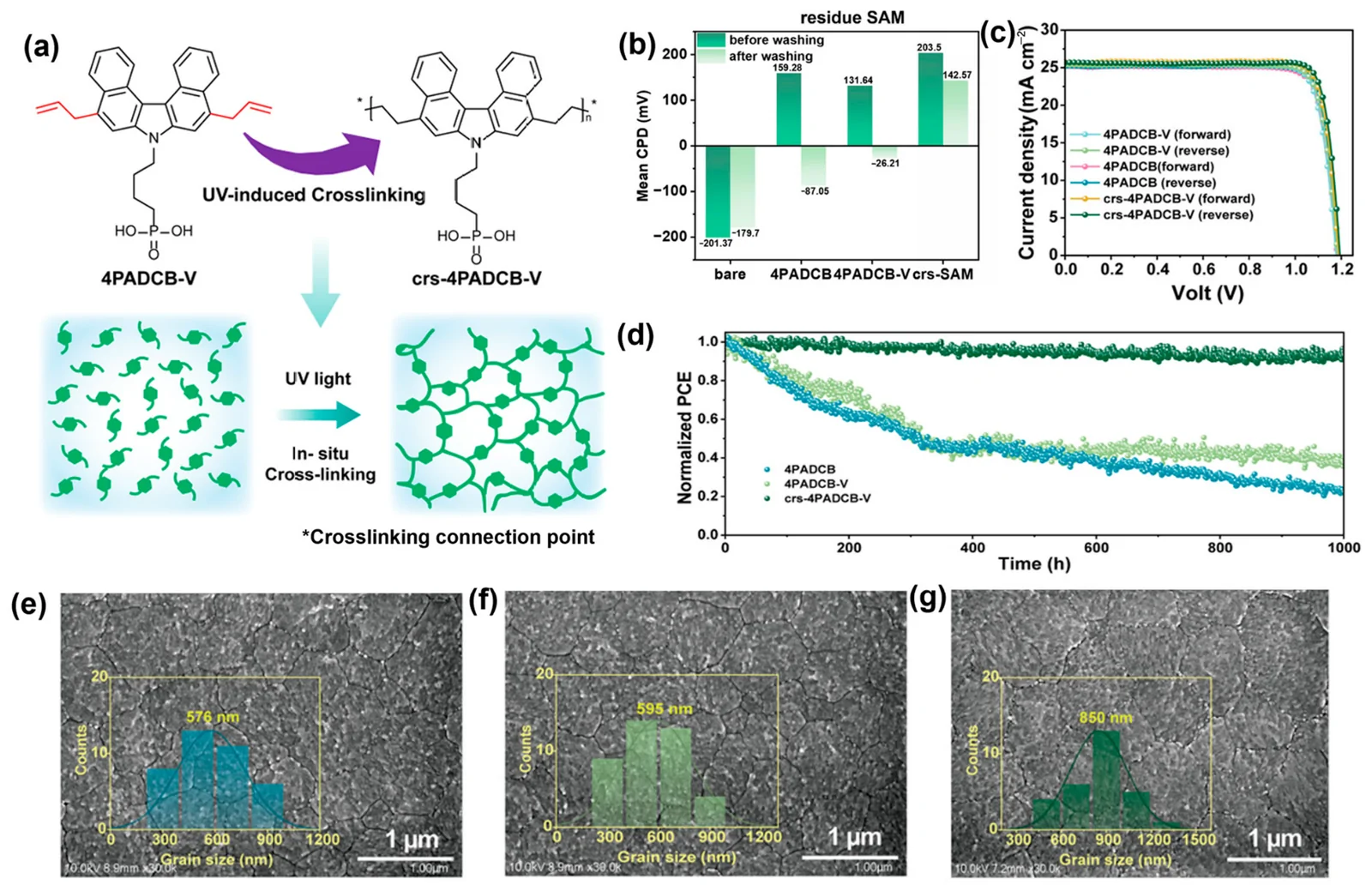
Molecular design, processing stability, and device performance of the in situ crosslinked SAM. (**a**) Molecular transformation of 4PADCB-V into crs-4PADCB-V through UV-induced crosslinking and schematic illustration of the in situ crosslinked SAM network, (**b**) mean contact potential difference (mean CPD) variation in different SAM-modified substrates before and after solvent washing, (**c**) J–V curves of inverted perovskite solar cells based on 4PADCB, 4PADCB-V, and crs-4PADCB-V, (**d**) normalized PCE evolution of devices with different SAMs under continuous illumination and maximum power point tracking, (**e**–**g**) SEM images and grain-size statistics of perovskite films deposited on 4PADCB, 4PADCB-V, and crs-4PADCB-V substrates, respectively. Reprinted with permission from Ref. [33]. Copyright 2025 John Wiley and sons.

**Figure 13:**
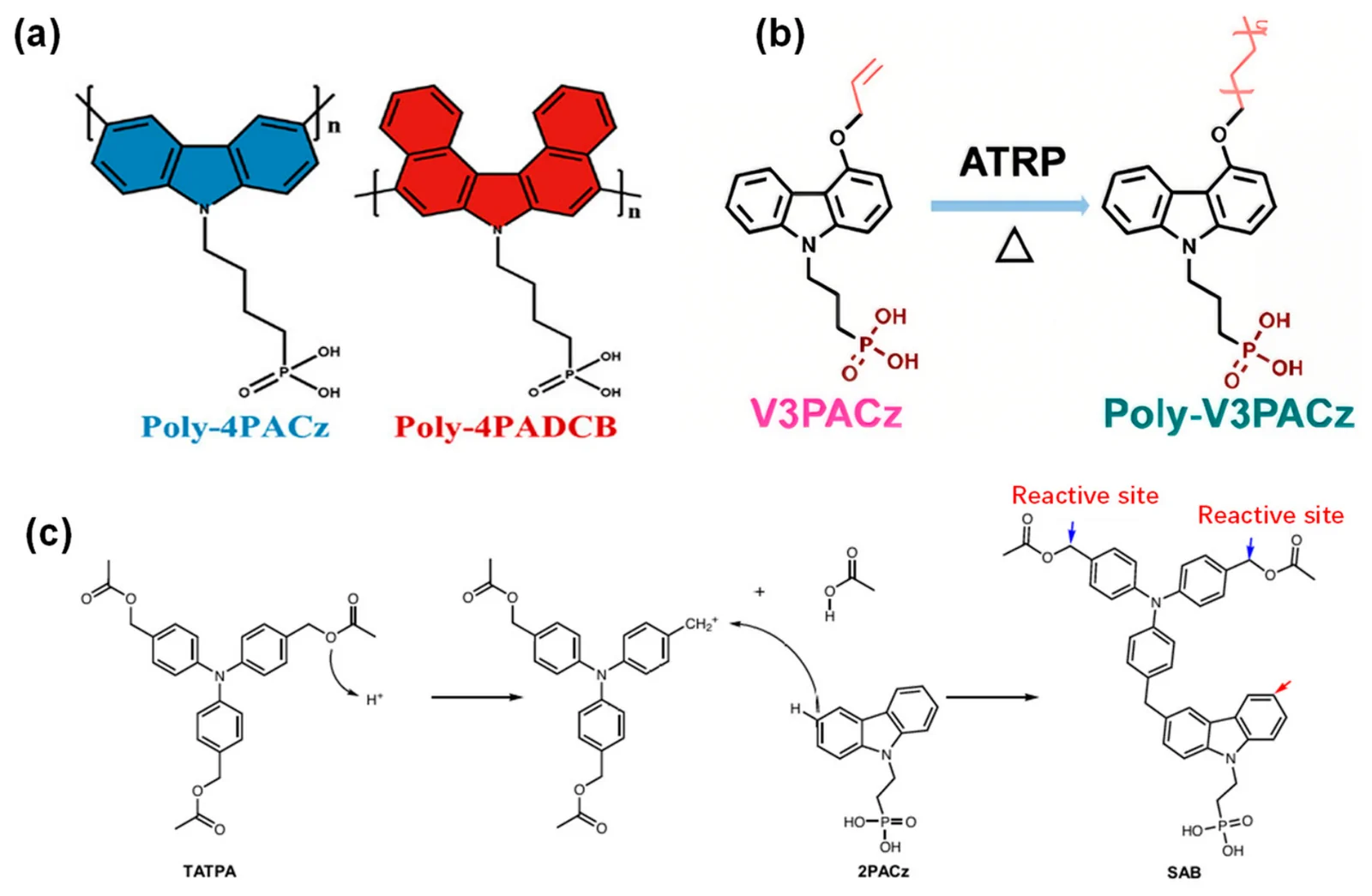
Representative polymerized and covalently reinforced SAM systems for inverted perovskite solar cells. (**a**) Molecular structures of Poly-4PACz and Poly-4PADCB, showing polymeric carbazole–phosphonic acid SAM analogues with different backbone/linkage structures. Reprinted with permission from Ref. [82]. Copyright 2025 John Wiley and Sons. (**b**) Polymerization of V3PACz to form Poly-V3PACz. Reprinted with permission from Ref. [81]. Copyright 2026 John Wiley and Sons. (**c**) Formation of a covalently linked self-assembled bilayer (SAB) through reaction between a triphenylamine-based layer and a phosphonic-acid SAM. Reprinted from Ref. [71].

Covalent crosslinking provides a direct route to polymerized SAMs by introducing intermolecular chemical linkages that reinforce the structural integrity and processing stability of the molecular layer. Wang et al. [33] developed an in situ crosslinked SAM strategy to overcome several limitations of conventional SAMs, including non-uniform coverage, weak interfacial adhesion and the desorption of weakly adsorbed molecules during polar-solvent processing. In this design, a dual-locking network is formed on the substrate surface by integrating molecule–substrate anchoring with molecule–molecule crosslinking, which improves the resistance of the SAM layer to subsequent perovskite solution processing. This reinforced interfacial network mitigates solvent-induced molecular desorption, interfacial rearrangement and coverage defects. As a result, crs- 4-(5,9-diallyl-7H-dibenzo[c,g]carbazol-7-yl)butyl phosphonic acid (4PADCB-V)-based devices achieved a maximum PCE of 26.46% and a certified PCE of 25.96%, while retaining 91% of their initial efficiency after 1000 h of maximum-power-point tracking under continuous illumination. These results suggest that in situ crosslinking is an effective strategy for simultaneously improving the processing stability of SAM interfaces and the operational stability of inverted PSCs (Figure 12). Similarly, Ling et al. [81] extended the in situ crosslinking concept to phosphonic-acid SAMs by designing 3-(4-(allyloxy)-9H-carbazol-9-yl)propyl)phosphonic acid (V3PACz) molecules with vinyl ether side groups (Figure 13b). Thermally induced in situ polymerization on ITO produced a Poly-V3PACz hole-selective layer with improved distribution uniformity, surface wettability and resistance to polar solvents. These features enhanced perovskite film quality and buried-interface contact. As a result, Poly-V3PACz-based devices achieved a maximum PCE of 25.21% and retained 92.1% and 91.9% of their initial efficiencies after 500 h of dark storage at 85 °C and 1200 h of MPP tracking, respectively. This work further supports in situ crosslinking as an effective approach for improving the processing stability of SAM interfaces and the long-term stability of inverted PSCs. Accordingly, covalent crosslinking transforms conventional SAMs from assemblies of relatively independent adsorbed molecules into ultrathin functional interfaces with lateral connectivity and network-like characteristics. This structural reinforcement improves coverage integrity, interfacial adhesion and operational stability while retaining the key advantages of SAMs, including energy-level modulation and hole selectivity.

Beyond intralayer crosslinking, bilayer self-assembly and covalent attachment of upper-layer molecules further extend the polymerized-SAM concept by introducing additional interfacial connectivity and structural reinforcement. Dong et al. [71]. developed a covalently linked self-assembled bilayer (SAB) strategy to mitigate molecular desorption and weakened interfacial contact in conventional hole-selective SAMs under thermal stress and temperature fluctuations (Figure 13c). In this design, a phosphonic-acid SAM was covalently interconnected with a triphenylamine upper layer through a Friedel–Crafts alkylation reaction, producing a robust polymeric network on the substrate surface. The resulting network withstood thermal treatment at 100 °C for 200 h, while the face-on-oriented triphenylamine upper layer strengthened adhesion with the perovskite and increased the interfacial adhesion energy by approximately 1.7-fold relative to a conventional SAM/perovskite interface. As a result, SAB-based inverted PSCs achieved a third-party certified PCE of 26.08%. Encapsulated devices exhibited less than 4% efficiency loss after 2000 h of damp-heat ageing at 85 °C/85% RH and less than 3% loss after 1200 thermal cycles between −40 and 85 °C, highlighting the effectiveness of bilayer SAM design in enhancing tolerance to thermal stress and thermal cycling. This SAB case highlights the potential of covalently linked bilayer SAMs to provide structural reinforcement against interface failure under high-temperature operation and thermal cycling, in addition to improving interfacial coverage integrity.

Polymeric SAM-derived contacts further extend this design concept by addressing the film discontinuity and narrow processing windows that often limit conventional small-molecule SAMs during large-area coating. Ren et al. [30]. reported poly(carbazole phosphonic acid) (Poly-4PACz), a polymeric analogue of PACz that retains phosphonic-acid anchoring groups and carbazole-based hole-transport units while providing improved film continuity, thickness tolerance and compatibility with scalable coating. Poly-4PACz-based p-i-n devices achieved a PCE of 24.4%, while 25.0 cm^2^ modules delivered an aperture-area efficiency of 20.7%. These results demonstrate that Poly-4PACz can combine the interfacial selectivity of PACz-based molecular contacts with the film-forming and processing advantages of a polymeric interfacial layer. Separately, Cen et al. [82]. developed Poly-4PADCB by optimizing the monomer linkage sites to reduce steric repulsion and promote a more favourable arrangement of the phosphonic-acid groups after polymerization (Figure 13a). This molecular design strengthened substrate anchoring and increased molecular coverage while improving the ordering of the polymeric interfacial layer. The Poly-4PADCB study therefore highlights the importance of polymer-backbone and linkage-site design in regulating anchoring-group accessibility, interfacial coverage and assembly behaviour.

Beyond Co-SAMs, amorphous SAMs and polymerized/crosslinked SAMs, several additional directions are gaining attention, including functionalized SAMs, composite interfaces and data-driven molecular design. Fluorinated SAMs use strongly electron-withdrawing substituents to tune interfacial dipoles, increase surface hydrophobicity and improve interfacial stability, and have been applied to dual-interface passivation in flexible PSCs [83]. Anchoring groups with multidentate binding capability, such as phosphonic acid groups, can bind to ITO/FTO surfaces through monodentate, bidentate or tridentate coordination modes, thereby strengthening the interfacial binding stability of SAMs on TCO substrates [84]. In addition, these anchoring groups may interact with perovskite precursors or buried-interface defect sites, influencing perovskite film formation and interfacial defect passivation. Together, these strategies show that SAM research is moving beyond single-molecule modification towards a broader interface-engineering paradigm that integrates functional terminal-group design, anchoring-structure optimization, composite-interface construction and data-driven molecular discovery.

### 4.4. Summary of Emerging SAM-Based Interface Engineering Strategies

Co-assembled, amorphous and polymerized or crosslinked SAMs address different limitations of conventional small-molecule monolayers. Co-SAMs provide functional complementarity and a broader tuning space for interfacial energetics, wettability and defect passivation [29]. Amorphous molecular contacts can reduce interfacial heterogeneity and promote more homogeneous perovskite growth [31]. Polymerized or crosslinked SAMs strengthen coverage integrity, resistance to polar-solvent processing and operational stability through intermolecular covalent connectivity [33]. These SAM strategies exhibit distinct advantages and limitations rather than forming a simple performance hierarchy. Conventional small-molecule SAMs provide molecularly thin and low-loss hole-selective contacts [18], but their coverage uniformity is strongly influenced by the hydroxyl density and surface chemistry of the oxide substrate [61]. Weakly adsorbed or multilayer molecules may also be unstable during subsequent solution processing [69]. Co-SAMs broaden the interfacial tuning space by combining complementary functions such as energetic regulation, wettability improvement and defect passivation [29], but their performance depends on precise control of component ratios, competitive adsorption, deposition sequence and spatial distribution [76]. Amorphous molecular contacts can suppress excessive molecular aggregation and promote more homogeneous perovskite growth [31]. However, amorphization must be achieved while preserving suitable molecular packing, charge selectivity and transport capability [79]. Polymerized or crosslinked SAMs improve coverage integrity and resistance to polar-solvent processing [33]; whereas, the additional polymerization, crosslinking or bilayer-assembly steps may increase fabrication complexity and process-control requirements [69]. Therefore, the reported advantages and disadvantages of different SAM strategies should be interpreted in relation to substrate chemistry, molecular structure, deposition route, device architecture and stability-testing conditions, rather than being attributed solely to the SAM category.

However, the performance of Co-SAMs depends strongly on the component ratio, deposition sequence and interface-formation kinetics [76]. For amorphous molecular contacts, molecular disorder must be balanced with charge-selective transport and structural stability; orthogonal π-conjugated skeletons demonstrate that amorphization can improve resistance to external stresses without sacrificing charge selectivity [79]. Covalently reinforced interfaces may also require additional crosslinking, reaction or bilayer-assembly steps, thereby increasing process-control requirements [71]. These approaches should therefore be regarded as complementary rather than universally superior strategies, with their suitability depending on device architecture, substrate properties, deposition route and targeted stability requirements.

## 5. Challenges and Perspectives

Despite the rapid progress of SAM-based inverted PSCs, several interconnected challenges remain before molecular-scale interface engineering can be translated into stable, reproducible and scalable photovoltaic technologies. These challenges mainly involve the understanding and control of SAM formation on practical substrates, large-area processing and reproducibility, dynamic characterization of buried interfaces, and predictive molecular design. Accordingly, the following discussion is organized around these four aspects.

### 5.1. Understanding and Controlling SAM Formation on Practical Substrates

Although SAMs have enabled effective buried-interface regulation in inverted PSCs, how they actually form on practical substrate surfaces remains insufficiently understood. SAM formation is often described using a simplified model in which molecules diffuse from solution to ITO, FTO or NiO_x_ surfaces, bind through anchoring units such as phosphonic acid or carboxylic acid groups to surface hydroxyl groups or metal sites, and subsequently orient into a monolayer. In practical devices, however, these substrates are far from ideal flat surfaces. Surface roughness, grain orientation, spatial variations in hydroxyl density and cleaning residues can all influence SAM adsorption density, molecular tilt angle, surface coverage and packing uniformity [21,35,61]. Kralj et al. [21] found that the microstructure of TCO substrates influences the work-function shift induced by 2PACz-SAM anchoring, leading to micro- and nanoscale heterogeneity in the work-function distribution. Similarly, Fu et al. [61] emphasized that phosphonic-acid SAMs preferentially react with surface hydroxyl groups on metal oxides to form P-O-M bonds, indicating that the hydroxyl density of the substrate is a key factor governing SAM adsorption strength and deposition uniformity. These microstructural variations can propagate into changes in interfacial dipoles, surface energy, precursor wetting and perovskite nucleation behaviour. At the same time, weakly bound or multilayer-adsorbed SAM molecules may desorb or be washed away during subsequent polar-solvent processing, which can perturb interfacial energetics and accelerate device degradation [69]. Looking ahead, integrating in situ ellipsometry, in situ infrared spectroscopy, in situ X-ray photoelectron spectroscopy (XPS), Atomic force microscopy (AFM)/Kelvin probe force microscopy (KPFM) and molecular dynamics simulations will be important for resolving the formation pathway of SAMs from solvated molecules to stable interfacial monolayers. Such combined experimental and computational approaches could help establish structure-interface-device correlations that link SAM structural parameters with interfacial energetics, perovskite film formation and device performance.

### 5.2. Preparation and Deposition Methods of SAM-Based Contacts

The preparation of functional SAM contacts generally involves substrate pretreatment, molecular deposition and post-deposition treatment. Because SAMs are molecularly thin, variations in substrate cleaning, solution composition, deposition conditions and thermal treatment can substantially affect molecular adsorption, surface coverage, interfacial energetics and subsequent perovskite film formation.

Substrate pretreatment is particularly important for phosphonic-acid-based SAMs because their anchoring depends on reactive hydroxyl groups on oxide surfaces. Fu et al. showed that H_2_O_2_/UV treatment introduced hydroxyl seeds on FTO and promoted more homogeneous SAM deposition [61]. Molecular-solution formulation is also important because solvent composition, concentration and aggregation state influence the competition between solution-phase aggregation and surface adsorption. For example, suppression of 2PACz aggregation improved molecular distribution and scalable device performance [72].

Spin coating remains the most widely used laboratory-scale deposition method owing to its simplicity and compatibility with rapid molecular screening. However, SAM coverage is sensitive to spin speed and solution-flow dynamics [42], while material utilization and compatibility with continuous manufacturing remain limited [43]. Immersion and soak-coating methods provide simpler and more material-efficient alternatives. Recent rapid soak-coating strategies have shortened SAM deposition time while enabling dense molecular coverage and extension to large-area and flexible devices [85]. Blade and slot-die coating are more compatible with scalable solution processing. Precisely controlled blade coating has been used to regulate the deposition sequence and interfacial functions of Co-SAM contacts [76]. Direct slot-die deposition of SAM molecules has also been demonstrated as a continuous and precisely metered coating route [86]. Nevertheless, these methods require careful control of solution delivery, coating speed and substrate wettability because local undercoverage in ultrathin SAMs may be difficult to detect through conventional thickness measurements. Vacuum evaporation provides a solvent-free alternative and avoids solution-wetting limitations. Evaporated carbazole-based SAM contacts can preserve favourable interfacial energetics and hole selectivity [87]. However, this route requires molecules with sufficient volatility and thermal stability, and the evaporation temperature, deposition rate and nominal thickness must be controlled to avoid molecular decomposition or unintended multilayer formation [88].

Post-deposition rinsing and thermal treatment can remove weakly adsorbed molecules and stabilize the molecular layer. For molecules containing reactive groups, UV- or thermally induced crosslinking can further improve resistance to subsequent polar-solvent processing [33,81]. Overall, the preparation route should be selected according to molecular solubility or volatility, substrate surface chemistry, solution wettability, targeted device area and compatibility with subsequent perovskite deposition.

### 5.3. Large-Area Uniformity, Scalable Processing and Reproducibility

Large-area uniformity remains a critical challenge for translating SAM-based buried interfaces from small-area devices to module-level applications. Because SAMs are molecularly thin, local variations in molecular adsorption and coverage can lead to spatially non-uniform interfacial properties. Fu et al. [61] demonstrated that H_2_O_2_/UV-induced hydroxyl seeding on FTO strengthened phosphonic-acid anchoring and homogenized SAM deposition, highlighting the importance of substrate surface chemistry for large-area SAM uniformity. In addition, the microstructure of the underlying TCO can produce micro and nanoscale variations in SAM-induced work-function modulation, which may further contribute to energetic heterogeneity across large substrates [21].

The scalability of a SAM deposition route should therefore not be evaluated solely by its nominal coating area. Large-area processing must preserve uniform molecular coverage and anchoring density across the entire substrate [61]. Spatial uniformity of the SAM-induced interfacial energetics should also be considered because local variations in the substrate and molecular layer can affect the work-function distribution [21]. Moreover, resistance to subsequent perovskite solution processing is essential, as weakly adsorbed or multilayer SAM molecules may undergo desorption or rearrangement during solvent exposure [69]. Scale-up should therefore involve coordinated optimization of molecular structure, solvent, substrate surface chemistry and deposition conditions rather than the direct transfer of processing parameters established for small-area devices.

Accordingly, the assessment of SAM-based interfaces for module-level applications should move beyond the peak efficiency of individual small-area devices and include large-area uniformity, fabrication yield, batch-to-batch reproducibility and long-term operational consistency [89]. Spatially resolved KPFM can reveal local variations in SAM-induced surface potential and work-function modulation [21]; whereas, PL mapping can evaluate the spatial uniformity of the subsequently deposited perovskite film [31]. These measurements, together with contact-angle characterization and statistical analysis across multiple devices and fabrication batches, can provide a more reliable assessment of large-area interfacial uniformity. Scalable SAM processing should ultimately be judged by whether molecular coverage, interfacial energetics and device performance remain spatially uniform and reproducible over module-relevant areas.

From techno-economic and life-cycle perspectives, SAM contacts require very low material loading and offer potential for scalable and cost-effective processing [19]. However, techno-economic analyses of perovskite photovoltaics indicate that manufacturing cost is determined by the complete device stack and process flow, including material consumption, fabrication yield, equipment requirements, module efficiency and production scale [90,91]. Likewise, the energy payback time (EPBT) is generally evaluated at the complete cell or module level based on cumulative energy demand and electricity generation, rather than being assigned to an individual interfacial layer [92]. Therefore, the percentage cost and EPBT specifically attributable to the SAM contact cannot yet be reliably compared among the systems discussed in this review. Future studies should combine module-relevant SAM processing with standardized techno-economic and life-cycle assessments to determine whether the low material consumption, low-temperature processing and improved device lifetime enabled by SAM contacts ultimately translate into lower manufacturing costs and shorter EPBTs.

### 5.4. In Situ and Operando Characterization of Dynamic Buried Interfaces

Current understanding of SAM-based buried interfaces still relies heavily on ex situ and static characterizations before and after fabrication, including Ultraviolet Photoelectron Spectroscopy (UPS), XPS, AFM, contact-angle measurements and steady-state PL. Although these techniques provide valuable information on energy-level alignment, chemical composition, surface morphology and wettability, they mostly capture interfacial states after specific processing steps. As a result, they are insufficient for resolving the dynamic evolution of SAM interfaces under real device-operation conditions [68]. Under realistic processing and operating conditions, including illumination, electric fields, thermal stress, humidity and subsequent solvent exposure, SAM-based buried interfaces may undergo molecular rearrangement, desorption of weakly adsorbed molecules, interfacial-dipole evolution, ion accumulation and interfacial chemical reactions. Such dynamic changes can directly affect selective hole extraction, interfacial non-radiative recombination and long-term operational stability [33,69,71]. Conventional SAMs can suffer from non-uniform coverage and weak interfacial adhesion, making them vulnerable to subsequent perovskite processing. In particular, polar solvents used during perovskite deposition may desorb weakly adsorbed SAM molecules, thereby perturbing the buried interface and compromising interfacial stability [33]. These limitations indicate that the key challenge is no longer simply to characterize the interface before and after ageing, but to resolve when, where and how interfacial failure begins during processing and operation. Looking forward, a broader use of operando XPS, in situ Grazing-Incidence Wide-Angle X-ray Scattering (GIWAXS), in situ PL/EL mapping, Time-of-Flight Secondary Ion Mass Spectrometry (ToF-SIMS) depth profiling and bias-dependent KPFM will be important for tracking the evolution of SAM-based buried interfaces from structural, chemical, ionic and electrostatic perspectives. Compared with post-fabrication static characterization, these in situ and operando approaches can provide a more realistic picture of interfacial processes during device fabrication and operation. Such insights will be essential for clarifying how SAM-interface failure is coupled with device-performance degradation [68,93,94]. A combination of complementary characterization techniques is therefore required to establish reliable SAM–interface–device correlations. XPS and FTIR can reveal molecular anchoring, chemical bonding and structural evolution; UPS and KPFM can evaluate work-function shifts and local energetic heterogeneity; AFM, ellipsometry and contact-angle measurements can characterize morphology, ultrathin-layer formation and wettability; PL, TRPL and SPV can probe interfacial recombination and carrier-transfer dynamics; and ToF-SIMS can track ion redistribution and interfacial degradation. The combined use of these techniques across different length and time scales is essential for obtaining a comprehensive understanding of SAM-based buried interfaces.

In addition, standardized stability protocols and consistent reporting conditions are needed to distinguish intrinsic SAM-interface degradation from degradation caused by perovskite bulk instability, top-interface reactions or encapsulation failure.

### 5.5. Data-Driven Molecular Design and Predictive Interface Engineering

The development of SAM molecules has traditionally relied on empirical design principles and iterative experimental screening. However, the performance of SAM-based buried interfaces is controlled by a complex combination of molecular and interfacial descriptors, including dipole moment, adsorption energy, HOMO level, surface energy, solubility, molecular packing and interfacial stability. This multidimensional design space makes it difficult to achieve rapid multiparameter co-optimization through experimental trial-and-error alone [35,59]. In this context, Density Functional Theory (DFT) calculations, molecular dynamics simulations and machine-learning models offer powerful tools for predictively assessing key SAM descriptors, including interfacial dipoles, adsorption configurations, binding energies, energy-level alignment and film-forming propensity. By narrowing the candidate-molecule space before experimental validation, these approaches can substantially improve the efficiency of SAM molecular screening [95,96]. A representative example is the work of Zhang et al. [96], who used machine-learning-assisted screening to identify PAPzO as a buried-interface engineering molecule with stronger interfacial binding and more favourable energy-level alignment. PAPzO-based devices achieved a PCE of 26.04% and retained 91.24% of their initial efficiency after 1200 h of continuous maximum-power-point tracking. This example illustrates the potential of machine learning to accelerate the discovery of SAM-related interface molecules with balanced energetic and stability attributes. In the future, these approaches could be extended to more complex SAM systems, including Co-SAMs, amorphous SAMs and polymerized SAMs, where molecular composition, adsorption sequence and interfacial configuration are strongly coupled with device performance. By resolving these composition–assembly structure–performance relationships, data-driven methods could accelerate the transition of SAM research from empirical molecular screening towards predictive and efficient interface engineering [95,96].

## 6. Conclusions

This review shows that the performance of SAM-based hole-selective contacts is governed by the coupling of molecular structure, assembly behaviour and buried-interface properties rather than by energy-level alignment alone. Anchoring chemistry, backbone structure and interfacial functional groups influence molecular adsorption, orientation, surface coverage, precursor wettability, defect interactions and resistance to processing stresses. However, these effects are also strongly dependent on substrate chemistry and deposition conditions. Conventional, co-assembled, amorphous and polymerized or crosslinked systems should therefore be regarded as complementary approaches for addressing different limitations in interfacial coverage, energetic alignment, film formation and stability, rather than as universally superior solutions.

Future progress will depend on resolving SAM formation and evolution on realistic oxide substrates, achieving reproducible large-area deposition while preserving molecular coverage, orientation and interfacial energetics, and establishing standardized operando or quasi-in situ characterization and stability-assessment protocols. These efforts will be essential for linking molecular design with interface evolution under practical processing and operating conditions, and for determining whether improvements demonstrated in small-area devices can be reliably translated to large-area modules.

## Data Availability

No new data were created or analyzed in this study. Data sharing is not applicable to this article.
